# Pharmacomicrobiomics of Non-Antibiotic Drugs: Mechanisms and Clinical Consequences of Gut Microbiota Alterations

**DOI:** 10.3390/pharmaceutics18060651

**Published:** 2026-05-26

**Authors:** Caterina Nela Dumitru, Alina Oana Dumitru, Larisa Goroftei, Elena Niculet, Mariana Daniela Ignat, Liliana Baroiu, Aurel Nechita, Gabriela Balan

**Affiliations:** 1 Research Centre in the Medical-Pharmaceutical Field, Faculty of Medicine and Pharmacy, “Dunărea de Jos” University of Galați, 35 Al. I. Cuza Street, 800010 Galați, Romania; 2 “Sf. Cuvioasa Parascheva” Clinical Hospital of Infectious Diseases, 800179 Galați, Romania; 3 Faculty of Medicine and Pharmacy, “Dunărea de Jos” University of Galați, 35 Al. I. Cuza Street, 800010 Galați, Romania; 4 “Sf. Ioan” Clinical Emergency Pediatric Hospital, 800487 Galați, Romania; 5 “Sf. Apostol Andrei” Emergency Clinical Hospital, 800578 Galați, Romania

**Keywords:** pharmacomicrobiomics, drug–microbiome interactions, gut microbiota, clinical pharmacology, drug metabolism, pharmacovigilance, proton pump inhibitors, metformin, oral iron, drug-induced dysbiosis, deprescribing

## Abstract

**Background**: The gut microbiota constitutes a metabolically active “second genome” that profoundly modulates drug pharmacokinetics, pharmacodynamics, and adverse reaction profiles. Beyond antibiotics, widely prescribed non-antibiotic pharmacotherapies exert clinically relevant pharmacomicrobiomic effects with implications for therapeutic optimisation and pharmacovigilance. **Methods**: This narrative review, conducted following PRISMA 2020 reporting principles (without PROSPERO pre-registration), searched PubMed/MEDLINE, Scopus, Web of Science, and Cochrane Library (January 2015–December 2024) for evidence on proton pump inhibitors (PPIs), metformin, NSAIDs, statins, SGLT2 inhibitors, and oral iron. Evidence tables included clinical human studies with molecular microbiota characterisation (16S rRNA or shotgun metagenomics), ≥20 participants, and a control arm; preclinical data informed mechanistic synthesis. **Results**: Of 68 eligible studies, 20 met criteria for the evidence tables. PPIs significantly remodelled gut microbiota composition with enrichment of oral-origin taxa (“oralisation of the gut”), associating with Clostridioides difficile infection and SIBO. Metformin enriched Akkermansia muciniphila and butyrate producers, contributing causally to glycaemic efficacy. NSAIDs compromised barrier integrity, with synergistic dysbiosis under PPI co-prescription. Statins correlated with reduced prevalence of the dysbiotic Bact2 enterotype. SGLT2 inhibitor data remained discordant. Oral iron consistently enriched Enterobacteriaceae at the expense of beneficial commensals.

## 1. Introduction

Pharmacomicrobiomics, the systematic investigation of bidirectional interactions between xenobiotics and the gut microbiota, has emerged over the past decade as a pharmacologically consequential dimension of drug action [1,2]. A concrete illustration from the panel of drugs analysed in the present review anchors the clinical relevance of the discipline: proton pump inhibitors (PPIs), among the most widely prescribed drugs globally, abolish the gastric acid barrier (with intragastric pH rising from <2.0 to 4.0–6.0) and permit the transit of oral-origin bacteria, continuously swallowed in saliva, to the colon, a phenomenon termed “oralisation of the gut” [3,4]. At the population level, the magnitude of this pharmacologically induced microbial perturbation exceeds that of any other dietary or lifestyle determinant [3,5], and its clinical consequences, increased risk of infection with *Clostridioides difficile*, small intestinal bacterial overgrowth, and community-acquired pneumonia, are now recognised as pharmacomicrobiomically mediated adverse drug reactions [6,7,8]. This example illustrates both the dominant direction of the interaction, a drug that reshapes the microbiota with downstream clinical consequences, and the role of the oral–gut axis as an anatomical–microbiological interface through which this remodelling is manifested [9]. The gut microbiota functions as a metabolically active “second genome” encoding enzymatic capabilities (β-glucuronidases, azoreductases, bile salt hydrolases, sulphatases, nitroreductases) that can activate prodrugs, inactivate parent compounds, regenerate biliary-excreted metabolites through enterohepatic recirculation, and generate microbially derived metabolites with off-target pharmacological activity [1,10,11]. Reciprocally, drugs reshape microbial ecology, altering the very ecosystem that modulates their pharmacokinetic and pharmacodynamic profile. This bidirectionality has direct consequences for drug efficacy, interpatient response variability, adverse drug reactions (ADRs), and rational pharmacotherapy.

The pharmacological relevance of this discipline is anchored in the metabolic capacity of the host–microbiota interface. The human gut microbiota encompasses an estimated 3.8 × 10^13^ bacterial cells, with the *Firmicutes* and *Bacteroidetes* phyla collectively accounting for approximately 90% of total microbial biomass under homeostatic conditions [12,13]. Beyond its endogenous physiological functions—including biosynthesis of essential vitamins (B12, K, folate), bile acid biotransformation, and short-chain fatty acid (SCFA) production with downstream GPR41/43 receptor signalling [14,15], this ecosystem operates as a parallel xenobiotic-metabolising compartment whose enzymatic repertoire substantially exceeds that of the human hepatic cytochrome P450 system. Disruption of microbial homeostasis (“dysbiosis”), characterised by reduced alpha diversity, loss of keystone taxa, and enrichment of potentially pathogenic microorganisms, has been mechanistically linked to inflammatory bowel disease, *Clostridioides difficile* infection (CDI), type 2 diabetes mellitus (T2DM), non-alcoholic fatty liver disease, and neuropsychiatric disorders via the gut–brain axis [16,17,18], pathologies that are themselves frequent indications for, or comorbidities of, the drug classes addressed in this review.

Drug-induced microbial perturbation is now recognised as a quantifiable pharmacological property of marketed compounds. Maier et al. (2018) screened 1079 approved drugs against 40 representative gut commensal strains and demonstrated that 24% of non-antibiotic compounds exhibit previously unrecognised antimicrobial activity at clinically achievable plasma concentrations, a finding with direct implications for drug safety profiling and ADR mechanism investigation [19]. At the population pharmacology level, Vich Vila et al. (2020), analysing metagenomic data from 1883 individuals with linked prescription records, identified 19 drug categories as showing significant single-drug associations with gut microbiome composition, with proton pump inhibitors (PPIs), metformin, NSAIDs, and statins producing the most robust signals [5]. The Flemish Gut Flora Project (n = 1106) independently confirmed medication use as a dominant covariate of microbiome variation, frequently exceeding the contribution of dietary, anthropometric, and lifestyle factors [20]. It must be emphasised, however, that these population-level associations are heavily influenced by polypharmacy and comorbidity: individuals receiving microbiome-active drugs frequently take several other agents and carry disease burdens that themselves alter microbial ecology, so the apparent signal of any single drug class in unadjusted analyses can be substantially confounded and is best interpreted alongside multi-drug models and mechanistic data. Collectively, these data establish drug exposure as a major determinant of inter-individual microbiome variability and, by extension, of microbiota-mediated pharmacological response.

The present review focuses on six drug classes, PPIs, metformin, NSAIDs, statins, SGLT2 inhibitors, and oral iron, because they jointly satisfy three selection criteria: (i) very high global prescription volume in adult ambulatory practice; (ii) reproducible off-target effects on microbial ecology demonstrated in independent human cohorts; and (iii) emerging mechanistic evidence that their therapeutic and/or adverse profiles are at least partly microbiota-mediated. Other microbiome-active drugs (e.g., antipsychotics, opioids, antineoplastics) fall outside this scope either because their indications are narrower, because their primary mechanism of microbiome interaction is already extensively reviewed elsewhere, or because the human evidence base remains too sparse to support clinically actionable inference. PPIs are among the most widely prescribed drugs globally; their primary mechanism, irreversible inhibition of the gastric H^+^/K^+^-ATPase, elevates intragastric pH from the bactericidal range (<2.0) to 4.0–6.0, abrogating the gastric acid barrier that normally restricts oral bacterial transit to the lower gastrointestinal tract [3,4]. Metformin (1,1-dimethylbiguanide), the cornerstone of T2DM pharmacotherapy prescribed to >150 million patients worldwide, has had its glucose-lowering mechanism substantially reinterpreted: a growing body of evidence demonstrates that a substantial proportion of its antidiabetic action is microbiota-mediated, specifically through modulation of *Akkermansia muciniphila* abundance and bile acid signalling pathways, repositioning the gut microbiota as a pharmacological effector compartment rather than a passive bystander [21,22]. NSAIDs inhibit cyclooxygenase isoenzymes with consequent prostaglandin depletion, disrupting gut mucosal defences and depleting anti-inflammatory commensals through mechanisms that may paradoxically perpetuate the mucosal inflammation these drugs are prescribed to attenuate—a textbook example of a microbiota-mediated ADR pathway [23,24].

Three additional drug classes complete the panel of clinically prioritised pharmacomicrobiomic interactions. Statins, 3-hydroxy-3-methylglutaryl-coenzyme A (HMG-CoA) reductase inhibitors prescribed to an estimated 145–200 million patients globally for cardiovascular disease prevention, modulate gut microbial composition through bile acid metabolism alteration and direct antimicrobial activity, with emerging evidence that part of their pleiotropic anti-inflammatory and metabolic benefits are microbiota-mediated [25,26]. SGLT2 inhibitors (empagliflozin, dapagliflozin, canagliflozin), now established in the management of T2DM, heart failure, and chronic kidney disease, alter the intestinal luminal environment through glycosuria-mediated substrate changes and have demonstrated favourable microbiome remodelling in recent clinical studies, although the evidence base remains preliminary [27,28]. Oral iron supplements, among the most commonly prescribed agents worldwide for iron deficiency anaemia, deliver unabsorbed ferrous iron to the colon, fundamentally altering microbial ecology by favouring siderophilic pathobionts (*Enterobacteriaceae*) at the expense of beneficial commensals, a pharmacological liability with direct implications for tolerability, mucosal inflammation, and clinical management strategies [29,30].

The concurrent prescription of multiple agents from this panel is prevalent in multimorbid patients, particularly elderly individuals with concomitant T2DM, cardiovascular disease, musculoskeletal disorders, and anaemia, generating complex polypharmacy scenarios with potentially compounding or counteracting pharmacomicrobiomic consequences. Such drug–drug–microbiome interactions extend the conventional pharmacological framework of drug–drug interactions (DDIs) by introducing a third variable: the microbial ecosystem that simultaneously metabolises and is reshaped by each co-administered agent. In parallel, microbiome-modulating co-interventions, including dietary fibre and polyphenolic compounds with documented prebiotic activity and gastrointestinal stability, have been proposed as pharmacological adjuncts to mitigate drug-induced dysbiosis and preserve therapeutic efficacy [31].

The aim of this review is to synthesise current clinical evidence (2015–2024) on the gut microbiota effects of six major non-antibiotic drug classes, proton pump inhibitors, metformin, NSAIDs, statins, SGLT2 inhibitors, and oral iron, with a focus on the pharmacological consequences for drug efficacy, safety, and adverse reaction profiles, and to derive translational implications for microbiome-aware prescribing, deprescribing, and adjunct co-therapy in polypharmacy contexts.

Beyond informing prescribing decisions, pharmacomicrobiomic insights are beginning to shape next-generation drug delivery. Microbiome-active drug delivery systems (MADDS) exploit predictable, microbiota-mediated transformations, such as bacterial β-glucuronidase, azoreductase or bile acid hydrolase activities, to trigger site-specific drug release in the colon and to tune local exposure on the basis of an individual’s microbial enzymatic repertoire [32]. Mapping which commensals are depleted or enriched by widely prescribed non-antibiotic drugs is therefore not only of pharmacovigilance interest but also a prerequisite for the rational design and patient stratification of such delivery platforms, and provides a forward-looking translational rationale for the present synthesis.

## 2. Materials and Methods

### 2.1. Study Design and Reporting

This work is presented as a narrative review applying PRISMA 2020 reporting principles [33] for methodological transparency, rather than as a formal systematic review with exhaustive enumeration of database records. The choice of a narrative format reflects a deliberate trade-off between methodological rigour and translational breadth: a formal systematic review with quantitative meta-analysis would have required restricting the synthesis to a single drug class with a relatively homogeneous evidence base (e.g., PPIs or metformin) and would have been undermined by substantial heterogeneity in microbiome characterisation methods, outcome definitions and reporting conventions across the six drug classes considered here. A narrative synthesis, by contrast, permits cross-class comparison and the integration of mechanistic, preclinical and pharmacovigilance evidence needed to derive clinically actionable prescribing implications, at the cost of greater reliance on author judgement in study selection and weighting. This trade-off was judged appropriate to the review’s translational aim, and methodological transparency has been preserved by reporting PICO criteria, search strategy, inclusion/exclusion rules and confidence grading explicitly. The protocol was developed a priori but was not pre-registered in PROSPERO; the unregistered protocol, search strategy, and data extraction forms are available from the corresponding author on reasonable request. Because of substantial heterogeneity in study designs, microbiome characterisation methodologies, and outcome definitions, a quantitative meta-analysis was not undertaken; a narrative synthesis with structured tabulation is presented. To maintain methodological rigour, the evidence tables presented in the Results section (one per drug class) were restricted to clinical studies in human participants, while mechanistic preclinical studies are discussed in the narrative text of each section but are not tabulated as primary evidence.

### 2.2. PICO Framework and Eligibility Criteria

The eligibility framework was operationalised according to the PICO structure (Table 1). Inclusion and exclusion criteria are specified in Table 2. Primary outcomes were changes in alpha and beta diversity and differential taxon abundance; secondary outcomes included CDI, SIBO, glycaemic parameters, intestinal permeability markers, cardiovascular endpoints, iron status parameters, and GI adverse events.

Reporting of diversity indices and bile acid measurements across the included studies was variable and is harmonised here according to the following principles. For alpha diversity, values are reported where available as Shannon index, observed ASVs/OTUs (richness), Chao1 or Faith’s phylogenetic diversity, with timepoints stated explicitly as baseline (pre-treatment), endpoint (post-treatment), or a within-subject delta where a paired pre/post design was used. For beta diversity, studies are described in terms of the distance metric used (Bray–Curtis, weighted or unweighted UniFrac) and the statistical test applied (most commonly PERMANOVA, occasionally multilevel PCA), with effect sizes (R^2^) reported where available. Where the original study reports diversity only at endpoint without a clear baseline, this is noted in the narrative and the finding is correspondingly treated as cross-sectional rather than longitudinal. For bile acid measurements, studies are distinguished by sampling compartment (faecal/intestinal versus plasma), assay approach (targeted UPLC-MS/MS panels versus untargeted UPLC-QTOF-MS metabolomics), and conjugation status reported (free, glycine- or taurine-conjugated, primary versus secondary). For example, Sun L. et al. (2018) [22] used targeted UPLC-QTOF-MS profiling in both intestinal/faecal and plasma matrices and explicitly reported conjugated species (GUDCA, TUDCA), whereas Freedberg et al. (2015) [34] reported faecal primary and secondary bile acid pools without plasma sampling. This matrix-, assay- and conjugation-resolved reporting is intended to prevent unwarranted equivalence across bile acid findings derived from heterogeneous methodologies.

### 2.3. Information Sources and Search Strategy

Searches were conducted in PubMed/MEDLINE, Scopus, Web of Science (Core Collection), and the Cochrane Library (CENTRAL), covering January 2015 to December 2024. The 2015 lower boundary reflects the standardisation of high-throughput 16S rRNA amplicon sequencing as the field methodology prerequisite for inclusion. The primary search string integrated three Boolean domains: (1) microbiome terminology; (2) drug exposure terms covering the six target drug classes; and (3) outcome terms covering diversity metrics, key taxa, SCFA and bile acid signalling. MeSH terms were applied in PubMed; equivalent controlled vocabularies were used in Scopus and Web of Science. Filters: human studies, English language. Reference lists of included articles were hand-searched (snowballing). The full Boolean string is reproduced unchanged from the original manuscript.

### 2.4. Study Selection and Data Extraction

Records identified through the structured searches were screened by the authors against the PICO criteria specified above; reference lists of relevant articles and prior reviews were hand-searched (snowballing) to identify additional eligible studies. Final inclusion decisions were reached by author consensus. Data extraction captured: author, year, country, study design, sample size, participant characteristics, drug agent/dose/duration, microbiota method (16S rRNA variable region, platform, pipeline, reference database), diversity metrics, differential taxa, and clinical outcomes. Seminal studies published before 2015 that were identified through snowballing and considered foundational to the field were included as exceptions to the temporal boundary.

## 3. Results

### 3.1. Study Selection and Overall Characteristics

Structured database searches and reference list snowballing identified 68 studies meeting the overall eligibility framework. Of these, 20 clinical studies in human participants form the core pharmacomicrobiomic evidence base presented in the drug-class-specific evidence tables in the Results section (five PPI, five metformin, two NSAID, three statin, two SGLT2i, three oral iron)the remaining 48 studies, comprising preclinical mechanistic investigations and prior systematic reviews, are integrated within the narrative synthesis to support causal inference and to clarify the molecular pathways linking drug exposure to microbial perturbation. Where multiple reports drew on overlapping or duplicate study populations (e.g., successive analyses of the LifeLines-DEEP cohort or repeat secondary analyses of the same RCT), only the most comprehensive or most recent primary report was retained in the evidence tables, with the related publications cited in the narrative as supporting analyses to avoid double-counting of participants. Of the 48 non-tabulated studies, the majority were preclinical mechanistic investigations, in vitro screens (e.g., anaerobic culture and high-throughput drug–commensal assays), rodent and germ-free or gnotobiotic mouse models, and faecal microbiota transplantation experiments, with prior systematic reviews and meta-analyses constituting a smaller fraction; narrative reviews without primary data were used only for contextual purposes and did not contribute taxonomic or outcome data to the synthesis.

**Figure 1:**
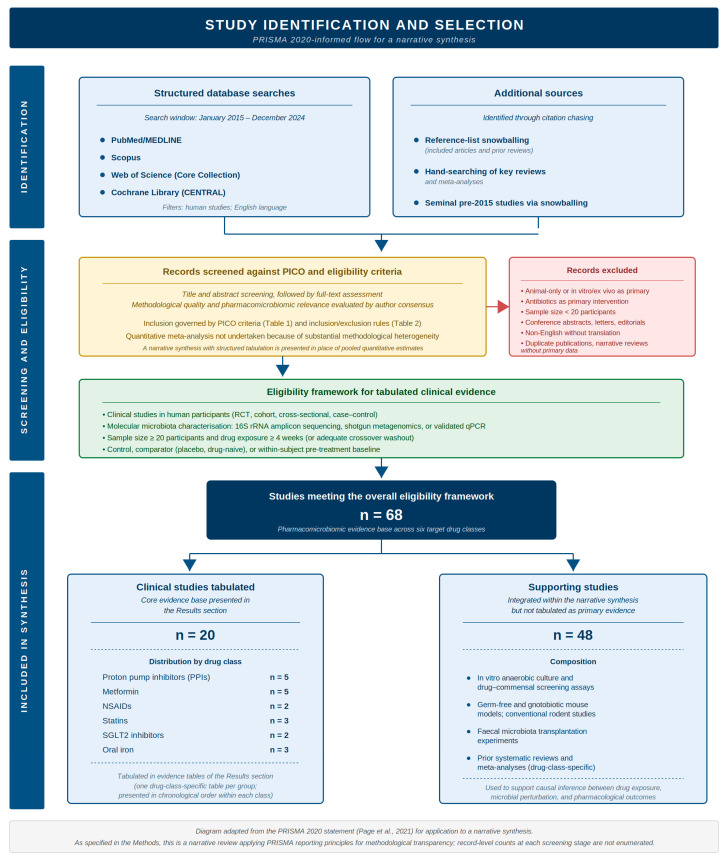
Study identification and selection flow, presented in a PRISMA 2020-informed format adapted for a narrative synthesis. As specified in Section 2.1, this work applies PRISMA 2020 reporting principles for methodological transparency rather than performing a formal systematic review with exhaustive enumeration of database records; consequently, the diagram displays the structure of the selection process rather than record-level counts at each screening stage [33]. Structured searches were conducted in PubMed/MEDLINE, Scopus, Web of Science Core Collection, and the Cochrane Library—CENTRAL (covering January 2015–December 2024, with filters for human studies and English language), and were complemented by reference-list snowballing of included articles and prior reviews, hand-searching of key reviews and meta-analyses, and identification of seminal pre-2015 studies through snowballing. Records were screened against the PICO framework (Table 1) and the inclusion/exclusion criteria (Table 2); methodological quality and pharmacomicrobiomic relevance were evaluated by author consensus. Because of substantial methodological heterogeneity across the included studies, a quantitative meta-analysis was not undertaken, and a narrative synthesis with structured tabulation is presented in its place. The applied eligibility framework retained clinical studies in human participants (RCT, cohort, cross-sectional, or case–control designs) characterised by 16S rRNA amplicon sequencing, shotgun metagenomics, or validated qPCR, with a sample size ≥ 20 participants and drug exposure ≥ 4 weeks (or an adequate crossover washout), and an appropriate control, comparator, or within-subject pre-treatment baseline. Sixty-eight studies met the overall eligibility framework. Of these, 20 clinical studies in human participants are tabulated as the core pharmacomicrobiomic evidence base in the Results section (proton pump inhibitors, n = 5; metformin, n = 5; NSAIDs, n = 2; statins, n = 3; SGLT2 inhibitors, n = 2; oral iron, n = 3). The remaining 48 supporting studies—encompassing in vitro anaerobic culture and drug–commensal screening assays, germ-free and gnotobiotic mouse models, conventional rodent dysbiosis studies, faecal microbiota transplantation experiments, and prior drug-class-specific systematic reviews and meta-analyses—are integrated within the narrative synthesis to support causal inference between drug exposure, microbial perturbation, and pharmacological outcomes. Abbreviations: PRISMA, Preferred Reporting Items for Systematic reviews and Meta-Analyses; PICO, Population–Intervention–Comparator–Outcome; RCT, randomised controlled trial; qPCR, quantitative polymerase chain reaction; rRNA, ribosomal RNA; PPI, proton pump inhibitor; NSAID, non-steroidal anti-inflammatory drug; SGLT2, sodium-glucose co-transporter 2. The figure was generated using Inkscape version 1.3 (https://inkscape.org, accessed on 4 April 2026).

Study designs of the included clinical studies encompassed cross-sectional analyses, prospective and retrospective pharmacoepidemiological cohorts, and randomised controlled trials, with sample sizes ranging from 20 to over 3000 participants. 16S rRNA amplicon sequencing was the predominant microbiota characterisation method, with shotgun metagenomic profiling employed in a subset of studies and combined approaches in others. Cohorts originated predominantly from European, Asian, and North American populations, reflecting the geographic distribution of pharmacomicrobiomic research infrastructure rather than the global epidemiology of the prescribing patterns under study. A schematic overview of study identification and selection is presented in Figure 1.

### 3.2. Methodological Quality Assessment

Methodological quality across the included clinical studies was qualitatively variable, with implications for the strength of pharmacological inference that can be derived from each drug class. Principal sources of potential bias identified by the authors include the predominance of cross-sectional designs in several drug classes (limiting causal attribution of microbial shifts to drug exposure), incomplete control of dietary patterns and concomitant medications (introducing residual confounding from co-administered xenobiotics that themselves exert pharmacomicrobiomic effects), reliance on self-reported drug use in observational pharmacoepidemiological cohorts (with potential misclassification of exposure duration and adherence), and substantial heterogeneity in microbiota characterisation methodology, including variable 16S rRNA hypervariable regions targeted, sequencing platforms, bioinformatics pipelines (with ongoing debate regarding OTU clustering versus exact amplicon sequence variant approaches (OTUs, operational taxonomic units, are sequence clusters grouped at a similarity threshold, typically 97%, whereas ASVs, amplicon sequence variants, are exact single-nucleotide-resolution sequence features generated by denoising algorithms such as DADA2; ASVs offer higher reproducibility across studies and are now generally preferred over OTUs)) [35,36], taxonomic reference databases, and analytical thresholds for differential abundance (most commonly assessed using LEfSe, the linear discriminant analysis effect size method that combines non-parametric testing with effect-size estimation to identify biomarker taxa, and DESeq2, a negative-binomial generalised linear modelling framework originally developed for RNA-seq and adapted for count-based microbiome data. Community-level differences between groups in this review are most often evaluated by PERMANOVA (permutational multivariate analysis of variance on a chosen distance matrix, such as Bray–Curtis or UniFrac), which tests whether between-group differences exceed within-group variation.

A foundational analytical caveat must be acknowledged when interpreting taxonomic shifts reported across the included studies. Microbiome sequencing data are inherently compositional: relative abundances are constrained to sum to unity within each sample, so an apparent “↑” or “↓” of a given taxon in a relative abundance space does not necessarily reflect a true ecological expansion or contraction of that taxon in vivo, but may instead arise from reciprocal changes in other community members. Consequently, all directional statements regarding ↑/↓ taxa in this review, across PPIs, metformin, NSAIDs, statins, SGLT2 inhibitors and oral iron, should be interpreted as compositional shifts rather than as confirmed absolute changes in microbial load, unless explicitly anchored by absolute quantification methods (e.g., quantitative microbiome profiling, QMP; RT-qPCR with reference standards; or flow cytometry-based microbial load estimates). Where such absolute or load-normalised data are available (notably the QMP-based MetaCardis statin analysis [25] and selected RT-qPCR studies of PPIs and oral iron [37,38]), they substantially strengthen ecological inference; conversely, observations restricted to relative abundance frameworks should be regarded as hypothesis-generating from an ecological standpoint, even when supported by robust statistical signals. The drug-class-specific evidence tables and accompanying narrative therefore distinguish, where possible, findings underpinned by absolute or load-normalised quantification from those derived from relative abundance approaches alone.

A related interpretive boundary applies to predicted functional outputs. Pathway-level inferences derived from PICRUSt and PICRUSt2, which extrapolate KEGG or MetaCyc functional content from 16S marker-gene data using reference-genome lookups, are at best hypothesis-generating and cannot substitute for direct measurement of microbial gene content or activity. In this review, statements such as “enrichment of bacterial invasion pathways” [34] or “methanogenesis and pyrimidine pathway shifts” [39] are accordingly treated as predicted functional patterns that warrant confirmation by shotgun metagenomics (for gene-level content) and/or metabolomics (for actual metabolic output), rather than as mechanistic proof. Findings that are corroborated by such orthogonal data, for example the metformin–SCFA axis, where 16S-predicted SCFA-pathway enrichment is supported by shotgun metagenomics and direct faecal SCFA measurement [21,40], are treated with greater interpretive weight than predicted-function-only observations.

The methodological heterogeneity acknowledged above warrants an explicit qualitative sensitivity assessment of how it may influence the directional consistency of taxa signals across drug classes. Targeted 16S hypervariable regions differ in their ability to resolve specific genera (e.g., V1–V2 amplicons under-represent *Bifidobacterium*, while V3–V4 and V4 provide more balanced coverage), so apparent inconsistencies in *Bifidobacterium* or *Akkermansia* signals across studies (e.g., discordant findings in some metformin and SGLT2i cohorts) may partly reflect amplicon choice rather than true biological variability. Differences in sequencing platforms (454 pyrosequencing vs. Illumina MiSeq/HiSeq) and read lengths further influence taxonomic resolution and rare-taxon detection. Bioinformatics pipelines (OTU clustering at varying similarity thresholds vs. ASV-based denoising; QIIME1 vs. QIIME2 vs. mothur; SILVA vs. GreenGenes vs. RDP reference databases) can yield different relative abundance estimates for the same biological samples, and analytical thresholds for differential abundance (LEfSe LDA cut-offs, FDR thresholds, prevalence filters) modulate which taxa surface as significant. Notwithstanding these sources of technical variability, the signals that emerge most consistently in this review—PPI-induced oralisation of the gut, metformin-induced *Intestinibacter* depletion and *A. muciniphila* enrichment, NSAID-associated *F. prausnitzii* depletion, statin-associated reduction in the dysbiotic Bact2 enterotype, and iron-induced *Enterobacteriaceae* expansion—reproduce across multiple combinations of amplicon region, platform, pipeline, and population, supporting their robustness to specific methodological choices. Class-specific findings reported only in single studies or with a single methodological pipeline are correspondingly treated as preliminary.

To translate these methodological considerations into a transparent appraisal of study strength, an informal but structured hierarchy for confounder control was applied when weighing the evidence for each drug class. Studies were considered most robust when they explicitly adjusted for, or matched on, four domains in combination: (i) dietary patterns (food-frequency questionnaire-based intake or, ideally, recent dietary records); (ii) anthropometric and metabolic confounders (BMI, glycaemic status, fasting lipids where relevant); (iii) recent antibiotic exposure (typically within 3–6 months prior to sampling, given documented prolonged effects of antibiotics on gut microbiota); and (iv) concomitant non-antibiotic medications known to be microbiome-active (PPIs, metformin, NSAIDs, statins, SGLT2i, oral iron, laxatives, and antipsychotics). Studies adjusting for all four domains, most notably the Vich Vila multi-drug analysis [5] and the Vieira-Silva MetaCardis statin analysis [25], were treated as the highest-quality observational evidence; studies adjusting for only one or two domains were treated as suggestive; and single-drug analyses without multi-drug adjustment were treated as preliminary, particularly where the index drug is commonly co-prescribed with other microbiome-active agents (e.g., NSAIDs in patients also receiving PPIs and antibiotics). This hierarchy is consistent with the limitations independently identified in the NSAID and polypharmacy sections, in which the loss of significance of the NSAID signal after multi-drug adjustment [5] is explicitly used to flag the importance of confounder control.

Causal inference language in this review draws on a pragmatic adaptation of Hill’s criteria, hereafter referred to as “modified Hill criteria”, applied uniformly across drug classes. The elements considered are: (i) temporality, demonstration that microbial shifts follow rather than precede drug exposure, ideally in a paired pre/post design; (ii) replication, reproducibility of directional findings across independent cohorts, populations and platforms; (iii) dose–response or exposure–response, a coherent relationship between drug dose, duration or systemic concentration and the magnitude of microbial change; (iv) experimental transfer, evidence from gnotobiotic colonisation, faecal microbiota transplantation, or monocolonisation experiments that the implicated microbial shift is sufficient to recapitulate the pharmacological phenotype in a recipient host; and (v) biological plausibility, a defined molecular mechanism (e.g., mucin secretion and Amuc_1100 signalling for *A. muciniphila*; bile salt hydrolase modulation for *B. fragilis*–FXR; loss of acid-mediated colonisation resistance for PPI-induced oralisation). Under this framework, the metformin evidence base satisfies all five elements; the PPI evidence base satisfies four of five (temporality, replication, exposure–response, plausibility), lacking formal experimental transfer of the dysbiotic phenotype to a naive host; the NSAID evidence base satisfies three of five (temporality, replication via observational cohorts, plausibility; with partial experimental support via germ-free indomethacin enteropathy models [41]); the statin and oral iron evidence bases satisfy two to three elements; and the current SGLT2 inhibitor evidence base, restricted to two discordant clinical trials and limited preclinical data, satisfies at most one to two. This explicit and uniform application of modified Hill criteria is intended to prevent selective causal framing across drug classes and to make the strength of causal inference traceable to specific evidential elements.

A further interpretive caveat concerns the anatomical compartment from which microbial data are derived. Most clinical studies reviewed here profile faecal samples, which predominantly reflect distal colonic luminal communities and only indirectly approximate the small bowel and mucosa-associated microbiota in which several of the most pharmacologically relevant phenomena occur, notably PPI-driven small intestinal bacterial overgrowth (SIBO) and NSAID-induced small intestinal injury. Studies that directly profile gastric mucosal, duodenal or jejunal aspirate samples (e.g., Shi et al. for PPI-treated GERD patients [42] and selected REIMAGINE-type small bowel mapping studies [43]) provide more anatomically appropriate evidence for proximal phenomena, but are far less common than stool-based cohorts. In the present review, statements about distal colonic dysbiosis are anchored predominantly in faecal data, whereas statements about SIBO, oralisation of the proximal gut, and NSAID enteropathy are interpreted as inferred from the combination of faecal compositional shifts (consistent with translocation/expansion of proximal-resident taxa) and the smaller body of direct small bowel/mucosal evidence, rather than as directly measured proximal observations. This compartmental distinction is made explicit in PPI, NSAID and polypharmacy narratives.

Finally, virulence- and pathogenicity-related language is reserved for studies that directly profile virulence determinants or clinically relevant strain-level features (e.g., qPCR of EPEC, ETEC, EHEC, *Salmonella* and *Clostridium* toxin genes in the Jaeggi and Paganini iron RCTs [30,44]; resistome profiling using ShortBRED in Vich Vila [5]), and is not used as a default interpretation of family- or genus-level 16S signals such as a relative increase in *Enterobacteriaceae* or *Streptococcaceae*. In particular, the term “pathobiont” is used in this review to describe taxa whose pathogenic potential is context-dependent and supported by at least genus-/family-level epidemiological linkage to enteric disease, while “pathogen” and “virulence factor” are reserved for studies with strain-level identification or direct profiling of virulence/toxin genes.

The reliance on 16S rRNA amplicon sequencing in most included studies, with shotgun metagenomics applied only in a subset, has direct consequences for how class-by-class findings should be interpreted. 16S profiling typically resolves bacterial composition to the genus level and, depending on the hypervariable region targeted and pipeline used, may misclassify or fail to discriminate closely related species; it also infers functional capacity only indirectly (e.g., via PICRUSt) and therefore provides limited insight into the microbial enzymatic activities of greatest pharmacological interest. Shotgun metagenomics, in contrast, supports species- and strain-level taxonomic resolution and direct profiling of drug-metabolising gene content (β-glucuronidases, azoreductases, bile salt hydrolases, antibiotic-resistance determinants), but is underrepresented in the included cohorts. Accordingly, drug classes whose evidence base derives mainly from 16S studies (notably SGLT2 inhibitors, NSAIDs and oral iron) should be interpreted as describing genus-level compositional shifts with predicted rather than measured functional consequences, whereas the metformin and PPI evidence, which includes shotgun metagenomic and gnotobiotic validation, supports stronger inferences about functional and species-level effects.

Confidence in the synthesised pharmacomicrobiomic evidence varies systematically across drug classes. Confidence is qualitatively highest for metformin, supported by multiple randomised controlled trials with mechanistic validation in germ-free murine models that satisfy modified Hill criteria for causal inference between drug-induced microbial shifts and pharmacodynamic outcomes. Confidence is similarly high for PPIs, supported by large, internally consistent observational cohorts demonstrating reproducible compositional shifts across geographically diverse populations. Confidence is intermediate for NSAIDs and statins, where directional signals are consistent across cohorts and the limited available RCTs, but the body of evidence is smaller and mechanistic validation in controlled experimental systems remains incomplete. Confidence is lowest for SGLT2 inhibitors and oral iron, reflecting the limited number of clinical studies with discordant or heterogeneous findings, and, in the case of SGLT2 inhibitors, the relative novelty of the drug class within pharmacomicrobiomic research. These confidence gradings are summarised in Table 3 and inform the strength of clinical translational recommendations developed in Section 3.3.

Across the included clinical studies, some broad patterns in confidence were apparent. Evidence quality was systematically higher in European and East Asian cohorts, reflecting the geographic concentration of pharmacomicrobiomic research infrastructure and access to shotgun metagenomic platforms; data from Latin American, African and South Asian populations were sparse and were dominated by the oral iron paediatric trials, limiting cross-population generalisability for the other five drug classes. With respect to age, the evidence base was strongest in middle-aged and older adults (in line with the typical indications for PPIs, metformin, statins, NSAIDs and SGLT2i) but weakest at the extremes, paediatric and frail elderly populations, with the notable exception of oral iron, where the most informative trials were specifically in infants and schoolchildren. By disease context, confidence was highest in metabolic disease (T2DM, obesity) and cardiovascular cohorts, intermediate in functional gastrointestinal and reflux populations, and lowest in patients with concomitant inflammatory bowel disease or advanced renal disease, where baseline dysbiosis and pleiotropic co-medication complicate drug-specific inference.

### 3.3. Proton Pump Inhibitors and Gut Microbiota

#### 3.3.1. Pharmacological Basis of PPI-Induced Microbial Perturbation

PPIs disrupt gut microbial ecology primarily by elevating intragastric pH from the bactericidal range (<2.0) to values consistently exceeding 4.0 during standard dosing and 6.0–7.0 with high-dose twice-daily regimens [45]. Under physiological conditions, the gastric acid barrier renders most swallowed oral bacteria non-viable within approximately 15 min, preventing the transit of ~10^8^ CFU/mL of salivary bacteria—corresponding to an estimated intake of ~10^9^ bacteria/day through the swallowing of approximately 1.5 L of saliva produced daily [9]—into the lower gastrointestinal tract [43]. PPI-mediated acid suppression abolishes this barrier, permitting viable oral-origin bacteria to reach the colon—a process termed “oralisation of the gut” [3,4]. Secondary pharmacological mechanisms include off-target inhibition of vacuolar H^+^/K^+^-ATPase in neutrophils and macrophages, impairing phagocytic killing capacity and further reducing mucosal colonisation resistance [46]. Reduced gastric acidity also impairs cobalamin liberation from food proteins, indirectly modifying the intraluminal nutrient milieu and niche availability for specific bacterial taxa [47]. These mechanisms position PPI-induced dysbiosis as an off-target pharmacological effect with direct relevance to the well-documented PPI-associated adverse drug reaction (ADR) profile.

#### 3.3.2. Clinical Evidence on Metformin-Induced Microbiota Modulation

Five clinical studies in human participants, conducted within the search window and meeting the eligibility criteria for the evidence tables, characterise the gut microbiota response to PPI exposure (Table 4). Findings are reinforced by a substantial body of complementary preclinical and small-cohort interventional data integrated within the narrative synthesis.

Causal validation in the absence of antibiotic confounding was provided by Freedberg et al. (2015) in an open-label crossover trial of 12 healthy volunteers receiving omeprazole 40 mg twice daily for 4 weeks following a 4-week baseline period [34]. Although overall faecal microbial diversity (Shannon index) remained unchanged on PPIs (*p* = 0.71), significant within-individual increases were observed in pre-specified taxa associated with *C. difficile* infection and gastrointestinal bacterial overgrowth: *Enterococcaceae* (*p* = 0.03), *Streptococcaceae* (>10-fold, *p* < 0.01), *Micrococcaceae* (*p* = 0.01), and *Staphylococcaceae* (*p* = 0.01), accompanied by a 44% median decrease in *Clostridiaceae* (*p* = 0.03). All taxonomic shifts in this study were quantified in a relative abundance space using 16S amplicon sequencing without absolute microbial-load normalisation, and should accordingly be interpreted as compositional rather than absolute-abundance changes. Functional metagenomic prediction (PICRUSt) revealed enrichment of pathways related to bacterial invasion of epithelial cells, a predicted-function output that is best treated as hypothesis-generating in the absence of shotgun-metagenomic or metaproteomic confirmation; faecal bile acid profiling (targeted analysis of luminal/faecal primary and secondary bile acid pools) showed no PPI-induced alterations in primary or secondary bile acids, suggesting that PPI-mediated CDI predisposition operates pharmacologically through reduced colonisation resistance rather than through altered intra-luminal bile acid signalling, with the caveat that plasma bile acid pools were not characterised in this study.

Imhann et al. (2016) applied 16S rRNA gene sequencing (V4 region) to 1815 participants spanning three Dutch cohorts, the LifeLines-DEEP general population study (n = 1174), an IBD cohort (n = 300), and an IBS case–control cohort (n = 341), and identified PPI use as exerting more prominent population-level pharmacomicrobiomic effects on the gut microbiome than antibiotics or any other commonly used drug class examined [3]. Meta-analysis revealed significant alterations in approximately 20% of detected bacterial taxa (FDR < 0.05), with consistent enrichment of oral-origin and potentially pathogenic genera, most notably *Rothia* (*p* = 9.8 × 10^−38^), *Streptococcus*, *Enterococcus*, *Staphylococcus*, *Veillonella*, and *Escherichia coli*, accompanied by a moderate but significant reduction in Shannon α-diversity. Principal coordinate analysis demonstrated a significant shift in the PPI-user gut microbiome toward an oral-cavity-like composition (PCoA1 *p* = 1.39 × 10^−20^), providing the first large-scale population pharmacology evidence for the “oralisation of the gut” phenomenon. Critically, the PPI-associated taxonomic shifts persisted after adjustment for antibiotics and concurrent medications, and showed substantial overlap with microbial signatures previously linked to *C. difficile* susceptibility (decreased *Bifidobacterium* and *Ruminococcaceae*; increased *Enterobacteriaceae*, *Enterococcaceae*, and *Lactobacillaceae*), establishing a pharmacomicrobiomic mechanism for the elevated enteric infection risk reported in clinical pharmacovigilance meta-analyses.

Independent replication and partial control for genetic confounding were provided by Jackson et al. (2016) in the TwinsUK cohort (n = 1827), where 16S rRNA profiling of faecal samples confirmed PPI-associated taxonomic shifts even after adjustment for shared genetic background through paired analysis of 70 monozygotic twin pairs discordant for PPI use [4]. Consistent with Imhann’s findings, PPI users showed significant enrichment of oral cavity commensals, most notably *Streptococcaceae* (q < 10^−6^, β = 0.46), *Micrococcaceae* (q < 10^−5^, β = 0.46), and the species *Rothia mucilaginosa* and *Streptococcus anginosus*, with concomitant depletion of beneficial gut commensals including *Lachnospiraceae* (q = 0.004, β = −0.35) and *Ruminococcaceae* (q < 0.001, β = −0.26), families with established roles in *C. difficile* colonisation resistance. Replication of the strongest associations in an independent interventional dataset strengthens causal inference under modified Hill criteria, although the cross-sectional core design precludes formal causal demonstration.

Hojo et al. (2018) [37] provided quantitative confirmation of PPI-induced dysbiosis using a complementary methodological approach. In a prospective pre–post study of 20 patients with reflux oesophagitis treated for 8 weeks (predominantly esomeprazole 20 mg), 16S/23S rRNA-targeted RT-qPCR—importantly, an absolute quantification approach that anchors directional inferences in microbial load rather than relative abundance—demonstrated significant increases in *Lactobacillus* spp., including the *L. gasseri*, *L. fermentum*, *L. reuteri*, and *L. ruminis* subgroups, as well as *Streptococcus*, *Enterobacteriaceae*, and *Staphylococcus*, while obligate anaerobic populations, total faecal organic acid concentrations, and luminal pH remained unchanged [37]. Notably, oral commensal streptococci (*S. salivarius*, *S. oralis*) were detected in the bloodstream of a subset of patients post-treatment, providing preliminary, though statistically non-significant, pharmacovigilance-relevant evidence consistent with the bacterial translocation hypothesis. This study is methodologically distinctive in that it quantified subdominant bacterial populations with high sensitivity using absolute quantification methodology, complementing rather than duplicating the relative abundance metagenomic evidence from larger cohorts [3,4].

Shi et al. (2019) extended these observations by directly contrasting the gastric mucosa-associated and faecal compartments in 55 GERD patients receiving omeprazole 40 mg/day, stratified into short-term (<1 year) and long-term (>1 year) users [42]. This study is particularly valuable because it samples a proximal mucosal compartment (gastric mucosa) directly, rather than inferring proximal microbial events from distal faecal data—an anatomically appropriate design for evaluating PPI-induced changes at the site of drug action. Using 16S rRNA gene sequencing (V4 region), they demonstrated that PPI exposure exerts compartment-specific pharmacomicrobiomic effects: gastric mucosal richness was significantly reduced in PPI users compared with non-PPI-user GERD patients, accompanied by enrichment of environmental and atypical taxa including *Planococcaceae*, *Oxalobacteraceae*, and *Sphingomonadaceae*. In the faecal compartment, α-diversity remained unchanged between PPI users and non-users, but compositional shifts were pronounced, with significant enrichment of oral-type *Streptococcaceae*, together with *Veillonellaceae*, *Acidaminococcaceae*, *Micrococcaceae*, and *Flavobacteriaceae*, mirroring the “oralisation” pattern reported in larger European cohorts [3,4]. The convergence of proximal (gastric mucosal) and distal (faecal) signals strengthens the inference that PPI-induced microbial perturbation involves both compartments, although it does not formally demonstrate translocation of specific taxa from the proximal to the distal compartment [48]. Notably, long-term PPI exposure was associated with progressive remodelling, evidenced by increased gastric Methylophilus and faecal Ruminococcus abundance relative to short-term users [42], supporting a duration-dependent dysbiotic trajectory of pharmacological relevance, recently extended at the functional level through the demonstration that the transcriptional activity of oral-origin species in the gut increases with the duration of PPI exposure [49].

#### 3.3.3. Supporting Mechanistic and Small-Cohort Evidence

Several smaller studies, while not meeting the strict ≥ 20-participant threshold for the evidence tables, provide important mechanistic complementarity. Mishiro et al. (2018) investigated the influence of esomeprazole 20 mg/day for 4 weeks on oral and gut microbiota in 10 healthy volunteers using 16S rRNA sequencing of faecal, salivary, and periodontal pocket fluid samples [50]. Salivary microbial Shannon diversity was significantly reduced after PPI exposure (*p* = 0.021), with PERMANOVA confirming significant β-diversity shifts in saliva (unweighted UniFrac *p* = 0.002, weighted UniFrac *p* = 0.015, Bray–Curtis *p* = 0.020) but not in faecal or periodontal pocket samples. At the genus level, *Streptococcus* abundance increased >4-fold in faecal samples (*p* = 0.044, q < 0.05), directly recapitulating the oralisation signature observed in larger cohorts—while *Neisseria* (*p* = 0.034, q < 0.05) and *Veillonella* (*p* = 0.008, q < 0.05) genera were significantly depleted in saliva. The observation of altered oral microbial composition extends the pharmacomicrobiomic footprint of PPIs beyond the gut and is mechanistically relevant to PPI-associated bacterial pneumonia risk through plausible aspiration of altered oropharyngeal flora, a hypothesis previously supported by historical case–control evidence including the seminal cross-sectional work of Lombardo et al. (2010) [51] on PPI-associated SIBO.

#### 3.3.4. Pharmacovigilance Implications

In framing the pharmacovigilance signal, it is useful to distinguish two overlapping but conceptually separate categories of risk associated with PPI exposure. The first comprises outcomes for which a microbiome-mediated mechanism is plausible and supported by convergent compositional data, chiefly *Clostridioides difficile* infection, SIBO, intestinal colonisation with multidrug-resistant organisms, and, by extension via aspiration of altered oropharyngeal flora, and community-acquired bacterial pneumonia, all of which align mechanistically with the PPI-induced loss of colonisation resistance and oralisation of the gut. The second comprises outcomes that remain only epidemiologically associated with PPIs and for which non-microbiome pathways (e.g., impaired calcium and B12 absorption, hypomagnesaemia, hypergastrinaemia-driven effects) are equally or more plausible—notably hip fracture in long-term users and several proposed cardiovascular and renal associations. Maintaining this distinction matters for interpretation: a microbiome-aware deprescribing strategy is most defensible for the first category, whereas the second supports general prudence in chronic PPI use without invoking microbiota as the mechanistic link. Pharmacovigilance meta-analyses confirm the clinical sequelae of PPI-associated microbial perturbations: pooled OR 1.99 (95% CI 1.73–2.30) for *C. difficile* infection (Trifan et al., 2017 [6]), with advanced age, comorbidity burden, and concurrent pharmacological acid suppression identified as independent predictors of CDI-associated mortality [52]; pooled OR 2.28 for SIBO (Lo and Chan, 2013 [7]); and pooled OR ~1.7 for multidrug-resistant organism colonisation (Willems et al., 2020 [53]). Immunocompromised patients are at disproportionately elevated risk, as attenuated mucosal immune surveillance compounds the colonisation resistance disruption attributable to acid suppression [6,53]. Beyond enteric complications, PPI-associated dysbiosis may contribute to extra-intestinal ADRs, including an increased risk of community-acquired pneumonia (pooled OR ~1.5; Lambert et al., 2015 [8]), a plausibly microbiome-linked outcome via altered oropharyngeal flora and hip fracture in long-term users [54], which by contrast is best regarded for now as an epidemiological association whose pathway through the microbiome remains speculative. Collectively, these data establish PPI exposure as a clinically actionable pharmacomicrobiomic ADR pathway warranting microbiome-aware deprescribing strategies in high-risk populations.

### 3.4. Metformin and Gut Microbiota

#### 3.4.1. Pharmacological Basis of Metformin–Microbiome Interaction

Metformin’s pharmacomicrobiomic action operates through three partially independent but functionally convergent pathways. First, selective enrichment of *Akkermansia muciniphila*, a Gram-negative anaerobic *Verrucomicrobium* constituting 0.5–5% of gut bacteria in healthy adults [55], occurs through metformin-driven upregulation of enterocyte mucin secretion (mediated by intestinal AMPK activation), expanding the mucosal niche selective for this bacterium’s specialised glycan metabolism [56]. *A. muciniphila* strengthens intestinal tight junction integrity via its surface protein Amuc_1100 and TLR2-mediated anti-inflammatory signalling, contributing to the metabolic phenotype that parallels metformin’s pharmacodynamic effects [56,57]. Second, a metformin-induced reduction in *Bacteroides fragilis* abundance increases intestinal levels of glycoursodeoxycholic acid (GUDCA), a bile acid that antagonises intestinal farnesoid X receptor (FXR) signalling and improves glucose homeostasis, a gut–liver–bile acid axis mechanism causally validated by Sun L. et al. (2018) [22,58]. Third, preclinical work by Bauer et al. (2018) in rats demonstrated metformin-induced modulation of upper small intestinal *Lactobacillales* that enhances duodenal SGLT1-mediated glucose sensing, potentiating GLP-1 secretion and vagal afferent suppression of hepatic glucose output, a gut–brain axis mechanism operating in parallel with the bile acid pathway [59]. Together, these mechanisms reposition metformin as a drug whose pharmacodynamic profile cannot be fully explained without reference to the gut microbiota as an active pharmacological effector compartment.

The microbiota-dependent enhancement of GLP-1 secretion described by Bauer et al. is of particular contemporary relevance given the rapid global expansion of the GLP-1 receptor agonist (GLP-1RA) prescribing for T2DM and obesity. If metformin’s glycaemic and weight-related actions are partly explained by upper-small-intestinal microbial modulation of endogenous GLP-1 release, then patients receiving combined metformin and GLP-1RA therapy plausibly experience convergent stimulation of the same enteroendocrine–vagal axis, with the microbiota acting as a shared upstream effector compartment. Emerging clinical pharmacology data are consistent with such an interaction, indicating that microbiome composition modulates the pharmacokinetic and pharmacodynamic response to GLP-1RAs and that dietary and microbial co-factors influence the magnitude of clinical effect [60,61]. These observations support a research priority of explicitly profiling microbiota in trials of metformin–GLP-1RA combinations and of considering microbiome-active dietary co-interventions as candidate enhancers of GLP-1RA efficacy, paralleling the strategies developed for metformin itself.

#### 3.4.2. Clinical Evidence on Metformin-Induced Microbiota Modulation

Five clinical studies in human participants, conducted within the search window and meeting the eligibility criteria for the evidence tables, characterise the gut microbiota response to metformin exposure (Table 5). Findings are reinforced by mechanistic preclinical validation in germ-free murine models and by recent evidence synthesis.

Forslund et al. (2015) performed the first systematic disentanglement of metformin’s microbiome signature from T2DM-associated dysbiosis using whole-genome shotgun metagenomics in 784 participants spanning Danish, Swedish, and Chinese cohorts (199 T2DM patients, 554 controls), with independent 16S rRNA validation in 30 additional patients [21]. The metformin-specific signal, enrichment of *Escherichia* spp. and depletion of *Intestinibacter*, was statistically independent of T2DM status, demonstrating that a substantial fraction of what had been attributed to T2DM-driven dysbiosis in the prior literature was confounded by metformin exposure. A dose–response correlation between microbial signature and serum metformin concentration provided pharmacological coherence, while functional metagenomic enrichment of butyrate (MF0114) and propionate (MF0125) biosynthesis modules suggested an SCFA-mediated mechanistic link to intestinal gluconeogenesis. Critically, the increase in *Escherichia* spp. correlated with the well-documented gastrointestinal ADR profile of metformin, providing a candidate pharmacomicrobiomic mechanism for tolerability variation.

Wu et al. (2017) conducted the first adequately powered double-blind placebo-controlled RCT specifically designed to assess metformin’s pharmacomicrobiomic action, randomising 40 treatment-naïve T2DM patients (22 metformin + 18 placebo) to 1700 mg/day metformin (titrated from 425 mg/day in the first week) or placebo for 4 months, with whole-genome shotgun metagenomic profiling of 131 faecal samples [40]. The metformin signature replicated Forslund’s findings (↑ *Escherichia*, ↓ *Intestinibacter*) and additionally identified a significant peak-to-trough ratio increase for *Bifidobacterium adolescentis* (negatively correlated with HbA1c, Spearman ρ = −0.28, *p* < 0.01), with targeted analysis confirming *A. muciniphila* enrichment after 4 months. Faecal propionate, butyrate, and lactate concentrations increased significantly under metformin, paralleling enrichment of bile salt hydrolase (bsh) genes and shifts in plasma bile acid profiles. Crucially, faecal microbiota transplantation from metformin-treated donors to germ-free mice causally transferred glucose tolerance improvement, providing the first formal Hill criteria validation of microbiota-mediated metformin pharmacology [40]. In vitro gut simulator experiments further demonstrated direct metformin effects on bacterial gene expression, with metallo-protein-encoding genes constituting the predominant regulated category, identifying microbial metalloproteins as putative direct pharmacological targets relevant to drug optimisation [40].

Sun L. et al. (2018) [22] advanced mechanistic understanding through a translational clinical study in 22 treatment-naïve T2DM patients (paired pre/post 3-day metformin 1000 mg b.i.d.), combining metagenomics with bile acid metabolomics, specifically, targeted UPLC-QTOF-MS profiling of conjugated and unconjugated primary and secondary bile acids in both intestinal/faecal and plasma matrices, enabling matrix-resolved comparison of bile acid signalling and validation in intestine-specific knockout mice, faecal microbiota transplantation, and *Bacteroides fragilis* monocolonisation experiments [22]. The largest species-level shift, a marked decrease in *B. fragilis*, was detectable within 3 days of metformin initiation, accompanied by accumulation of the conjugated secondary bile acids GUDCA and TUDCA (with concordant changes detectable in both intestinal/faecal and plasma compartments, supporting a systemic rather than purely luminal effect), suppression of intestinal FXR signalling (↓ FGF19, ↑ C4), and reduced bile salt hydrolase activity of *B. fragilis* [22]. The study defined the *B. fragilis*–GUDCA–intestinal FXR axis as a central pharmacomicrobiomic mediator of metformin action, identified GUDCA as the first endogenous human FXR antagonist (functional analogue of murine TβMCA), and identified BSH inhibition and FXR antagonism as druggable targets emerging from metformin pharmacomicrobiomics [22,62,63].

De la Cuesta-Zuluaga et al. (2017) extended these findings to a non-European, non-Asian Latin American cohort, conducting a cross-sectional 3:1 matched analysis (sex, age, BMI) of 112 Colombian adults: 14 T2DM-met^+^, 14 T2DM-met^−^, and 84 non-diabetic controls, with 16S rRNA sequencing (V4 region) and LEfSe biomarker analysis [64]. Compared with metformin-untreated T2DM patients, metformin users exhibited a 3.4-fold enrichment of *A. muciniphila* (*p* = 0.003, q = 0.01) and increased relative abundance of SCFA-producing taxa (*Butyrivibrio*, *Bifidobacterium bifidum*, *Megasphaera*, and a *Prevotella* OTU). Notably, T2DM patients without metformin showed enrichment of *Clostridiaceae* 02d06 and depletion of *Enterococcus casseliflavus*. By replicating the metformin signature in a population with distinct dietary patterns and baseline microbial composition, this study established the cross-population validity of metformin’s pharmacomicrobiomic effect and reinforced the rationale for considering metformin a microbiome-active drug across diverse genetic and dietary backgrounds—a finding with implications for global pharmacovigilance generalisability.

**Table 5:** Clinical studies characterising the gut microbiota response to metformin exposure in human participants.

Author, Year	Design	N	Agent & Duration	Key Microbiota Finding	Pharmacological/Clinical Consequence
Forslund et al., 2015 [21]	Multi-cohort cross-sectional metagenomics (Danish, Swedish, Chinese) + 16S rRNA validation cohort	784 (199 T2DM, 554 controls; +30 independent validation)	Metformin 500–2000 mg/day; chronic prevalent use	Metformin-specific signal statistically independent of T2DM status: ↑ *Escherichia* spp., ↓ *Intestinibacter* (consistent across three country cohorts). Dose–response correlation between microbial signature and serum metformin concentration. Functional metagenomic enrichment of butyrate (MF0114) and propionate (MF0125) biosynthesis modules in metformin-treated patients.	First systematic disentanglement of metformin’s microbiome signature from T2DM-associated dysbiosis, demonstrated that a substantial fraction of previously reported T2DM dysbiosis was confounded by metformin exposure. *Escherichia* enrichment correlated with GI ADR profile, providing a candidate pharmacomicrobiomic mechanism for metformin tolerability variation. SCFA-mediated mechanistic link to intestinal gluconeogenesis suggested by butyrate/propionate module enrichment.
Wu et al., 2017 [40]	Double-blind placebo-controlled RCT + FMT to germ-free mice + in vitro gut simulator (SHRIM)	40 treatment-naïve T2DM (22 metformin, 18 placebo); 131 faecal samples	Metformin 1700 mg/day (titrated from 425 mg/day, week 1); 4 months	Replicated Forslund signature: ↑ *Escherichia*, ↓ *Intestinibacter*. Significant ↑ peak-to-trough ratio of *B. adolescentis* (negatively correlated with HbA1c: Spearman ρ = −0.28, *p* < 0.01). ↑ *A. muciniphila* at 4 months (targeted analysis). ↑ faecal propionate, butyrate, lactate. ↑ bile salt hydrolase (*bsh*) gene abundance; shifts in plasma bile acid profiles. In vitro gut simulator: metformin regulated metalloprotein-encoding genes as predominant category in *A. muciniphila* and *B. wadsworthia*.	First adequately powered RCT specifically designed to assess metformin’s pharmacomicrobiomic action. FMT from metformin-treated donors to germ-free mice causally transferred glucose tolerance improvement, first formal Hill criteria validation of microbiota-mediated metformin pharmacology. Identification of microbial metalloproteins as putative direct pharmacological targets relevant to drug optimisation.
Sun et al., 2018 [22]	Translational clinical study (paired pre/post) + metagenomics + bile acid metabolomics + gnotobiotic mouse validation + monocolonisation experiments	22 treatment-naïve T2DM (paired pre/post 3-day metformin); validated in *Fxr*ΔIE, *Ampkα1*ΔIE knockout mice	Metformin 1000 mg b.i.d.; 3 days	Largest species-level shift: marked ↓ *B. fragilis* within 3 days of metformin initiation. Accumulation of conjugated secondary bile acids GUDCA and TUDCA. Suppression of intestinal FXR signalling (↓ FGF19, ↑ C4). ↓ *B. fragilis* BSH activity and *Bsh* gene copy number.	Defined the *B. fragilis*, GUDCA, intestinal FXR axis as a central pharmacomicrobiomic mediator of metformin action. Identified GUDCA as the first endogenous human FXR antagonist (functional analogue of murine TβMCA). Identified BSH inhibition and FXR antagonism as druggable targets emerging from metformin pharmacomicrobiomics.
De la Cuesta-Zuluaga et al., 2017 [64]	Cross-sectional, 3:1 matched analysis (sex, age, BMI); 16S rRNA sequencing (V4) + LEfSe biomarker analysis	112 Colombian adults (14 T2D-met^+^, 14 T2D-met^−^, 84 non-diabetic controls)	Metformin as prescribed; chronic prevalent use	T2D-met^+^ vs. T2D-met^−^: 3.4-fold ↑ *A. muciniphila* (*p* = 0.003, q = 0.01). ↑ SCFA-producing taxa: *Butyrivibrio*, *Bifidobacterium bifidum*, *Megasphaera*, and a *Prevotella* OTU. T2D-met^−^ vs. controls: ↑ *Clostridiaceae* 02d06; ↓ *Enterococcus casseliflavus*. β-diversity: significant for metformin users vs. non-users (PERMANOVA R^2^ = 0.013, *p* = 0.036); no significant α-diversity differences.	Replicated the metformin–microbiota signature in a non-European, non-Asian Latin American cohort with distinct dietary patterns and baseline microbial composition. Established cross-population validity of metformin’s pharmacomicrobiomic effect, reinforcing the rationale for considering metformin a microbiome-active drug across diverse genetic and dietary backgrounds. Implications for global pharmacovigilance generalisability of microbiome-mediated metformin effects.
Petakh et al., 2023 [65]	PRISMA 2020-compliant systematic review (6 RCTs, 4 quasi-experimental, 3 cross-sectional; 2301 records screened)	13 clinical studies included	Metformin various doses (425–2000 mg/day); durations 3 days—12 months	Consistent genus-level changes: ↓ *Intestinibacter* (four independent cohorts). ↑ *Blautia*, *Butyrivibrio*, *Escherichia*, *Bilophila*, *Bifidobacterium* (≥2 studies each). Inconsistent findings: *Akkermansia* (↑ in 2, ↓ in 2 studies); *Bacteroides* (↑ in 1, ↓ in 2). α- and β-diversity outcomes inconsistent across populations.	Confirmed reproducibility of the core *Intestinibacter*-depletion/SCFA-producer-enrichment signature across heterogeneous designs, doses, sequencing platforms, and populations. Methodological heterogeneity precluded definitive quantitative synthesis. Highlighted the need for standardised multicentric protocols and in vitro microbiome–drug screening assays to advance pharmacomicrobiomic methodology.

Abbreviations and symbols: ↑, increase/enrichment relative to comparator or baseline; ↓, decrease/depletion relative to comparator or baseline.

#### 3.4.3. Supporting Evidence Synthesis

A recent PRISMA 2020-compliant systematic review by Petakh et al. (2023) synthesised evidence from 13 clinical studies (six RCTs, four quasi-experimental, three cross-sectional) screened from 2301 records [65]. Genus-level changes proved consistent across studies, with *Intestinibacter* depletion confirmed in four independent cohorts and *Blautia*, *Butyrivibrio*, *Escherichia*, *Bilophila*, and *Bifidobacterium* enrichment in ≥2 studies each, while diversity outcomes (α and β) remained inconsistent. Methodological heterogeneity in design, dose, sequencing region, and population precluded definitive quantitative synthesis but reinforced the reproducibility of the core *Intestinibacter*-depletion/SCFA-producer-enrichment signature. The review highlighted the need for standardised multicentric protocols and in vitro microbiome–drug screening assays to advance pharmacomicrobiomic methodology [65].

#### 3.4.4. Pharmacotherapeutic Implications

Metformin exemplifies a pharmacological paradigm in which a drug’s therapeutic and adverse profiles are partly mediated by induced microbial shifts. Microbiome-modulating co-interventions, including dietary fibre and polyphenolic compounds with documented prebiotic activity, may potentiate metformin’s microbiota-mediated antidiabetic effects through synergistic A. muciniphila enrichment, representing a promising avenue for tolerability optimisation and dose-sparing strategies [31,66,67,68]. Pharmacogenomic variation in OCT1-mediated intestinal metformin uptake further modulates the extent of intraluminal drug exposure available for microbiota interaction, contributing to inter-individual variability in both efficacy and GI tolerability [69]. The reproducible association between Escherichia enrichment and gastrointestinal ADRs offers a candidate predictive biomarker for individualised metformin pharmacotherapy, Table 5.

### 3.5. NSAIDs and Gut Microbiota

#### 3.5.1. Mechanisms of NSAID-Induced Microbiome Disruption

NSAIDs disrupt gut microbial ecology through COX-dependent and COX-independent mechanisms. COX-1/2 inhibition suppresses PGE2 and PGI2, prostaglandins that maintain mucus secretion, bicarbonate release, mucosal blood flow, and epithelial tight junction integrity [24,70]. Prostaglandin depletion thins the mucus layer, the primary physical habitat of mucosal-adherent commensals, and increases transepithelial permeability to luminal antigens [71], creating a microenvironment favouring aerotolerant and proteolytic taxa at the expense of obligate anaerobes. COX-independent mechanisms include direct mitochondrial uncoupling in enterocytes, enterocyte apoptosis via p53-dependent pathways, and local reactive oxygen species generation [72], all of which promote focal epithelial disruption selectively colonised by pathobionts.

A pharmacologically important distinction must be drawn between aspirin and the non-selective NSAIDs. Non-selective NSAIDs (ibuprofen, naproxen, diclofenac, indomethacin) typically deplete butyrogenic, anti-inflammatory commensals such as *Faecalibacterium prausnitzii* and *Roseburia* spp. and enrich *Enterobacteriaceae*, a pattern consistent with COX-1/2 inhibition, prostaglandin-mediated mucus loss and barrier disruption. Aspirin, by contrast, presents a distinct microbiological profile: at antiplatelet doses it has been associated, somewhat paradoxically, with relative enrichment of *Bifidobacterium* and *A. muciniphila* and with overall preservation of α-diversity in several cohorts [73]. Several non-mutually exclusive mechanisms have been proposed: irreversible COX-1 acetylation with comparatively limited disruption of intestinal prostaglandin pools at low doses, anti-inflammatory effects of salicylate metabolites on mucosal oxidative stress, and direct microbial metabolism of aspirin by gut bacteria that may reduce its luminal bioavailability while shaping community composition [73]. Clinically, this distinction implies that the aspirin–microbiome profile cannot be assumed to generalise to non-selective NSAIDs and supports treating aspirin separately in pharmacomicrobiomic syntheses and in prescribing guidance.

#### 3.5.2. Clinical Evidence on NSAID-Associated Microbiota Alterations

Rogers and Aronoff (2016), in a cross-sectional observational study of 155 adults from a Michigan community cohort (62 NSAID users), demonstrated that drug type influenced microbiome composition more than the number of drugs taken, with molecule-specific NSAID-associated microbial profiles and consistent depletion of *Faecalibacterium prausnitzii*, *Roseburia* spp., and reduced *Firmicutes/Bacteroidetes* ratio across ibuprofen, diclofenac, indomethacin, and naproxen studies [23]. *F. prausnitzii* is of particular significance: constituting up to 5% of healthy gut bacteria and producing butyrate with potent anti-inflammatory properties [74,75], its depletion creates a pharmacological paradox in which anti-inflammatory drug therapy perpetuates mucosal pro-inflammatory conditions by eliminating a key endogenous anti-inflammatory regulator. Xiao et al. (2017) established causality using germ-free mouse models, demonstrating that gut microbiota composition critically modulates indomethacin-induced enteropathy severity, and faecal microbiota transplantation from healthy donors substantially attenuates indomethacin-induced small intestinal injury, providing direct mechanistic evidence that the gut microbiota mediates protection against NSAID-induced enteropathy [41].

The PPI + NSAID combination warrants special attention: Wallace et al. (2011) demonstrated that co-administration of PPIs with NSAIDs produced synergistic dysbiosis and small intestinal injury exceeding either drug alone, paradoxically undermining the gastroprotective rationale for co-prescription by compounding small intestinal microbiome damage in the very compartment that PPIs do not pharmacologically protect [76,77]. Importantly, this synergy was demonstrated using direct microbial profiling of the small intestine in a rodent model, providing anatomically appropriate evidence for the proximal compartment in which NSAID-induced enteropathy occurs [76]; analogous direct small-intestinal profiling in humans remains rare, and most clinical inferences about PPI + NSAID enteropathy are extrapolated from faecal microbiome data combined with capsule endoscopy or biomarker-based indicators of mucosal damage such as faecal calprotectin [78]. At the population level, Vich Vila et al. (2020) documented dose-dependent depletion of *Roseburia intestinalis* by NSAIDs (r = −0.41, *p* < 0.001) in 1883 individuals in a single-drug analysis [5]; notably, after correction for polypharmacy in multi-drug models, NSAIDs did not remain among the dominant-impact drug categories (PPIs, metformin, antibiotics, and laxatives retained the strongest independent signals), indicating that NSAID–microbiome associations in clinical practice are substantially confounded by co-prescribed medications [5]. Table 6 summarises key NSAID studies.

### 3.6. Statins and Gut Microbiota

#### 3.6.1. Mechanistic Basis of Statin–Microbiome Interactions

Statins interact with the gut microbiome through at least three identified pathways. First, HMG-CoA reductase inhibition alters hepatic bile acid synthesis by reducing cholesterol availability as a precursor, modifying the composition of the bile acid pool delivered to the intestine; since bile acids serve as potent antimicrobial agents selectively shaping gut microbial ecology, alterations in their profile directly impact bacterial community structure [25,58]. Second, certain statins, particularly simvastatin and atorvastatin, exhibit direct in vitro antimicrobial activity against Gram-positive species at physiologically achievable intestinal concentrations, as demonstrated by Maier et al. (2018), who identified statins among non-antibiotic drugs with significant growth-inhibitory effects on commensal bacteria [19]. Third, statins modulate systemic and intestinal inflammation through pleiotropic anti-inflammatory mechanisms (NF-κB suppression, endothelial function improvement), which indirectly influence the gut microenvironment and the competitive balance between pro-inflammatory and anti-inflammatory bacterial taxa [26,79]. Additionally, statin-induced changes in intestinal cholesterol availability modify bacterial membrane biosynthesis and may selectively favour cholesterol-assimilating taxa [80].

#### 3.6.2. Clinical Evidence on Statin-Associated Microbiota Changes

Vieira-Silva et al. (2020) provided a landmark population-level analysis, examining the gut microbiome of 2004 individuals from three independent Belgian cohorts using 16S rRNA sequencing [25]. Notably, this study applied quantitative microbiome profiling (QMP), in which 16S sequencing data are normalised by flow-cytometry-derived microbial loads to yield estimates of absolute rather than relative abundance—a methodological feature that substantially strengthens ecological interpretation by avoiding the compositional artefacts inherent in standard relative abundance pipelines. Statin use was associated with a partial correction of obesity-linked dysbiosis: statin users exhibited higher representation of *Bacteroidales* and anti-inflammatory *Faecalibacterium*, with attenuation of the *Firmicutes*-dominated obesogenic signature. Critically, this effect was independent of BMI, diet, and concurrent medication use, suggesting a direct pharmacomicrobiome mechanism. The authors proposed that statin-mediated microbiome modulation contributes to the drugs’ cardiovascular protective effects beyond lipid lowering.

In framing the statin signal as predominantly favourable, three converging ecological observations should be made explicit. First, statin therapy is associated with relative enrichment of SCFA-producing taxa, particularly Faecalibacterium and Roseburia [25], whose butyrate production supports colonocyte energetics, mucosal barrier integrity, and regulatory immune tone [81]. Second, statin exposure is linked to the reduced prevalence of the dysbiotic “Bact2” enterotype, dominated by Bacteroides and characterised by low microbial load, which is characteristic of obesity- and inflammation-associated dysbiosis [25], that is, a partial correction of obesogenic dysbiosis rather than a complete shift in community structure. Third, these compositional changes are accompanied by lower systemic inflammatory markers in observational data [82], consistent with a broader anti-inflammatory ecological shift. The favourable framing in this section refers to this combined pattern—enrichment of SCFA producers, attenuation of obesity-related dysbiotic configurations, and an ecological tone that reduces inflammation—rather than to any individual taxonomic change.

Khan et al. (2018), in a high-fat-diet rat model exploring atorvastatin’s effects across multiple doses, demonstrated that atorvastatin treatment increased gut bacterial diversity in the HFD group and modulated the relative abundances of *Oscillospira*, *Parabacteroides*, and *Ruminococcus* toward a normal-diet profile, providing preclinical mechanistic support for statin–microbiome interactions [83]. Kim et al. (2019), using an aged mouse model of HFD-induced obesity, reported that both atorvastatin and rosuvastatin increased the abundance of *Bacteroides*, *Butyricimonas*, and *Mucispirillum*, with these compositional shifts correlating with reduced ileal IL-1β expression and partial restoration of glycaemic control [84]. Liu et al. (2018) demonstrated, in an in vivo study, that gut microbiome composition is associated with the lipid-lowering efficacy of rosuvastatin, providing further mechanistic evidence for microbiota-mediated statin response variability [85].

Sun B. et al. (2018), in a comparative analysis of statin-response patients in East China, identified distinct gut microbial signatures associated with differential statin efficacy, suggesting that microbiota composition may modulate inter-individual variability in statin response [86]. Caparrós et al. (2021), in a comprehensive review of dysbiotic microbiota interactions in Crohn’s disease, highlighted *F. prausnitzii* depletion and *Enterobacteriaceae* enrichment as recurrent dysbiotic signatures in inflammatory bowel disease, providing context for understanding how anti-inflammatory pharmacological interventions, including statins, may interact with intestinal dysbiosis [87]. Hu et al. (2021), in a prospective single-centre cohort of 133 acute coronary syndrome (ACS) patients with a median 2.16-year follow-up, demonstrated that chronic statin therapy shifts the ACS gut microbiome toward a healthier intermediate state between ACS and healthy controls, with enrichment of butyrate- and propionate-producing taxa (*Bifidobacterium longum*, *Anaerostipes hadrus*, *Ruminococcus* (*Blautia*) *obeum*) and depletion of *Parabacteroides merdae* [39]. In vitro anaerobic co-culture in the same study confirmed direct atorvastatin–microbe interactions, supporting the gut microbiota as a co-target underlying statin pleiotropy in ACS. At the population level, Zhernakova et al. (2016), in the LifeLines-DEEP Dutch cohort (n = 1135 general-population participants, including 56 statin users among 44 drug categories analysed), identified statin use as among the medication classes most strongly associated with gut microbiota composition, contributing independent explanatory power to inter-individual Bray–Curtis variation [88]. This study thus established a general-population baseline against which clinical statin–microbiota effects can be interpreted, and provided the methodological framework subsequently adopted by the MetaCardis analysis [25]. Table 7 summarises key statin studies.

Taken together, the statin evidence base is characterised by a marked asymmetry: preclinical data, rodent models of high-fat-diet-induced dysbiosis, in vitro antimicrobial screens, and mechanistic bile acid studies substantially outnumber the available clinical pharmacomicrobiomic studies, which remain dominated by a small number of large observational cohorts and a limited number of intervention studies. As a result, the clinical evidence base for statins should not be regarded as comparably mature to that for metformin or PPIs, and the favourable inferences drawn here are appropriately framed as emerging rather than established, pending adequately powered RCTs with standardised microbiome profiling.

### 3.7. SGLT2 Inhibitors and Gut Microbiota

#### 3.7.1. Mechanistic Basis of SGLT2 Inhibitor–Microbiome Interactions

SGLT2 inhibitors (gliflozins) produce pharmacomicrobiome interactions through several postulated mechanisms. The primary mechanism involves glycosuric redistribution of glucose metabolism: by blocking renal glucose reabsorption, SGLT2i reduce circulating glucose levels while increasing urinary glucose excretion, which may indirectly alter the intestinal luminal substrate environment through modified enterocyte glucose flux and reduced systemic hyperglycaemia-driven mucosal inflammation [27]. SGLT2 is minimally expressed in the intestine; however, the related transporter SGLT1 is highly expressed in the proximal small intestine, and compensatory SGLT1 upregulation during SGLT2 inhibition has been demonstrated, potentially altering small intestinal glucose availability for bacterial fermentation [28,89]. Additionally, SGLT2i-mediated ketogenesis increases circulating β-hydroxybutyrate, a ketone body with immunomodulatory properties that may influence the gut mucosal immune environment and indirectly affect bacterial community composition [90]. Weight reduction and improved insulin sensitivity associated with SGLT2i therapy represent additional indirect pathways through which these drugs may modify the gut microbiome [27].

#### 3.7.2. Clinical Evidence on SGLT2 Inhibitor–Microbiota Interactions

Clinical evidence on SGLT2 inhibitor–microbiome interactions remains clearly preliminary and discordant across the two available RCTs meeting the inclusion criteria, and any inferences drawn at this stage must therefore be treated with caution. Deng et al. (2022) provided the strongest positive signal in a randomised, open-label, 3-month trial of 76 treatment-naïve T2DM patients with cardiovascular risk factors, comparing empagliflozin (10 mg/day, n = 40) with metformin (1700 mg/day, n = 36): empagliflozin uniquely improved cardiovascular risk factors and was associated with significantly reshaped gut microbiota composition, increasing short-chain fatty acid-producing bacteria (*Roseburia*, *Eubacterium*, *Faecalibacterium*) while reducing *Escherichia-Shigella*, *Bilophila*, and *Hungatella* [91]. The accompanying multi-omics correlations between microbial taxa and plasma metabolites (sphingomyelin, uric acid, cis-aconitate, glycochenodeoxycholate) are described in the original publication as associations; in the absence of formal mediation modelling or experimental transfer (e.g., FMT from empagliflozin-treated donors into germ-free or antibiotic-treated recipients), these relationships are best interpreted as “microbiota–metabolite associations consistent with”, rather than as evidence that microbial shifts “mediate”, the observed metabolic and cardiovascular phenotype. By contrast, van Bommel et al. (2020), in a double-blind randomised trial of 44 T2DM patients on background metformin assigned to dapagliflozin or gliclazide for 12 weeks, detected no significant effect of either treatment on gut microbiome alpha diversity or composition [92]—an important negative result indicating that not all SGLT2i effects reach clinical detectability at current sample sizes and treatment durations. An important translational caveat that may reconcile these divergent results is that background metformin therapy itself produces a strong, reproducible microbiome signature (in particular A. muciniphila enrichment, expansion of SCFA producers, and Intestinibacter depletion) [65], which may plausibly mask or saturate any class-specific incremental effect of an SGLT2 inhibitor added on top of it, consistent with the general principle that drug effects on the microbiome under polypharmacy are substantially confounded by co-medication that is microbiome-active [5]. The negative van Bommel result—obtained on stable background metformin—and the positive Deng result—obtained in treatment-naïve patients in direct comparison with metformin—are therefore not necessarily contradictory and may reflect this ceiling effect. Preclinical evidence suggests that dapagliflozin may produce subtle microbiota shifts, including trends toward increased *Akkermansia muciniphila* in db/db diabetic mice, accompanied by improvements in vascular function [28].

Mechanistic preclinical work complements this limited clinical evidence: germ-free rodent models have established that gut microbiota substantially modulates uremic solute accumulation relevant to the gut–kidney axis [93]; comparative SGLT2i trials such as PRIME-V (ipragliflozin vs. metformin, n = 103) [94] offer complementary pharmacological context without directly profiling the gut microbiome. Taken together, the current clinical evidence base for SGLT2 inhibitor–microbiome interactions is insufficient to support definitive recommendations; larger, adequately powered trials with standardised microbiome profiling—ideally including treatment-naïve arms or factorial designs that explicitly disentangle SGLT2i effects from background metformin—are required before SGLT2i can be reliably integrated into microbiome-informed pharmaceutical care. Table 8 summarises the two clinical studies meeting our inclusion criteria.

### 3.8. Oral Iron Supplements and Gut Microbiota

#### 3.8.1. Mechanistic Basis of Iron–Microbiome Interactions

Oral iron supplementation represents perhaps the most nutritionally direct pharmacomicrobiome interaction among the six drug classes reviewed. The bioavailability of standard oral iron formulations (ferrous sulfate, ferrous fumarate, ferrous gluconate) is inherently low (5–15%), meaning that 85–95% of ingested iron transits unabsorbed to the colon, where it fundamentally alters the bacterial competitive landscape [29,30]. Iron is an essential micronutrient for the majority of bacterial species, required for electron transport chain function, DNA replication, and oxidative stress defence; however, bacteria vary dramatically in their iron-acquisition capacity. Gram-negative *Enterobacteriaceae*, including *Escherichia coli*, *Salmonella*, and *Klebsiella*, possess highly efficient siderophore-based iron-acquisition systems (enterobactin, aerobactin, yersiniabactin) that confer a profound competitive advantage in high-iron environments [95,96]. It should be emphasised that the family- or genus-level enrichment of *Enterobacteriaceae* reported in iron supplementation studies does not, in itself, constitute evidence of pathogenicity: virulence and infectious disease relevance can only be inferred where strain-level identification or direct virulence/toxin-gene profiling has been performed (e.g., EPEC, ETEC and EHEC qPCR in the Jaeggi and Paganini RCTs [30,44]). Statements in this section regarding “pathobiont selection pressure” are accordingly anchored on those studies that explicitly profiled virulence determinants, and are not extrapolated from generic family-level shifts. In contrast, commensal *Lactobacillus* and *Bifidobacterium* species have evolved iron-independent metabolic strategies (using manganese-dependent enzymes) and lack siderophore systems, rendering them competitively disadvantaged when excess luminal iron is available [97].

Additionally, unabsorbed iron catalyses Fenton reactions generating reactive oxygen species (ROS) in the intestinal lumen, promoting oxidative stress that selectively damages strict anaerobes (which possess limited oxidative stress defence mechanisms) while favouring aerotolerant pathobionts [30,98]. Iron-mediated ROS production also contributes to mucosal inflammation and epithelial tight junction disruption, creating a feed-forward cycle of dysbiosis and barrier dysfunction [29].

#### 3.8.2. Clinical Evidence on Oral Iron–Induced Microbiota Dysbiosis

Jaeggi et al. (2015) conducted the first well-powered RCT examining iron supplementation effects on gut microbiota, randomising 115 Kenyan infants to iron-fortified micronutrient powder vs. iron-free control for 4 months [30]. Iron supplementation significantly increased *Enterobacteriaceae* (including pathogenic *Escherichia coli*) and *Clostridium* spp. while decreasing *Bifidobacterium* and *Lactobacillus*. Importantly, iron-supplemented infants showed elevated faecal calprotectin, a marker of intestinal inflammation, suggesting that iron-driven dysbiosis has functional pro-inflammatory consequences. Paganini et al. (2017) extended these findings in a factorial RCT (n = 155 Kenyan infants) demonstrating that co-supplementation of iron with galacto-oligosaccharide (GOS) prebiotic partially mitigated iron-induced *Enterobacteriaceae* expansion and preserved *Bifidobacterium* abundance [44], establishing proof-of-concept for combined iron-prebiotic nutritional strategies.

Zimmermann et al. (2010) conducted a 6-month RCT in 139 Ivorian schoolchildren aged 6–14 years randomised to iron-fortified biscuits (20 mg electrolytic iron, 4×/week) vs. unfortified control, demonstrating significant iron-induced compositional dysbiosis with increased enterobacteria and decreased lactobacilli, accompanied by elevated faecal calprotectin indicative of intestinal inflammation [38]. Dostal et al. (2014), using human gut microbiota-associated rats, demonstrated that iron supplementation significantly increased caecal acetate, propionate, and especially butyrate concentrations compared with iron-deficient rats, while iron deficiency caused major dysbiosis with decreased *Roseburia* spp./*Eubacterium rectale* and *Bacteroides* spp. abundance, directly linking luminal iron availability to microbial metabolic activity [99]. Lee et al. (2016), investigating neonatal iron status as a determinant of early-life gut microbiota and infant health outcomes, provided context for understanding how iron status during the perinatal period shapes subsequent microbiome development [100]. Kortman et al. (2014), in a comprehensive review integrating clinical and mechanistic data, proposed a model in which luminal iron excess creates a “pathobiont selection pressure” favouring virulence gene expression in *Enterobacteriaceae*, linking iron-induced microbiome changes directly to infection susceptibility [95]. Table 9 summarises key oral iron studies.

### 3.9. Polypharmacy and Drug–Microbiome Interaction Landscapes

The concurrent prescription of multiple drug classes in multimorbid patients creates complex pharmacomicrobiome scenarios not predictable from single-drug data. With the expanded six-drug-class analysis, 15 pairwise and 20 three-way combinations are theoretically possible—several have direct clinical prevalence and available evidence.

The PPI + NSAID combination produces synergistic dysbiosis and compounded small intestinal injury through converging mechanisms: PPI-induced Gram-negative enrichment in the proximal intestine amplifies NSAID-mediated barrier disruption in an anatomical site pharmacologically unprotected by acid suppression [76]. The Metformin + PPI scenario presents concurrent selective pressures with partial reciprocal attenuation [5], while Metformin + NSAID may offer partial protection through butyrate-mediated mucosal support [101]. Triple polypharmacy (PPI + Metformin + NSAID) represents a high-risk microbiome scenario, with a non-linear synergism of adverse signals potentially exceeding metformin’s protective microbiome modifications [76,102], a combination requiring dedicated prospective investigation.

The Statin + PPI combination, highly prevalent in elderly cardiovascular patients, presents a partially antagonistic pharmacomicrobiomic interaction: the beneficial statin-driven enrichment of F. prausnitzii and Roseburia [25] may partially compensate for the PPI-induced depletion of these same taxa [3], although the net clinical effect remains empirically uncharacterised. The Metformin + Statin combination, prescribed to a substantial proportion of T2DM patients with cardiovascular comorbidity, may produce synergistic beneficial microbiome effects, as both drugs independently enrich butyrate-producing taxa and Akkermansia [25,65,101], although formal studies are lacking.

The Metformin + SGLT2i combination, increasingly common in contemporary T2DM management, presents a particularly intriguing scenario given both drugs’ apparent enrichment of *A. muciniphila* through convergent but mechanistically distinct pathways [28,40,91].

**Figure 2:**
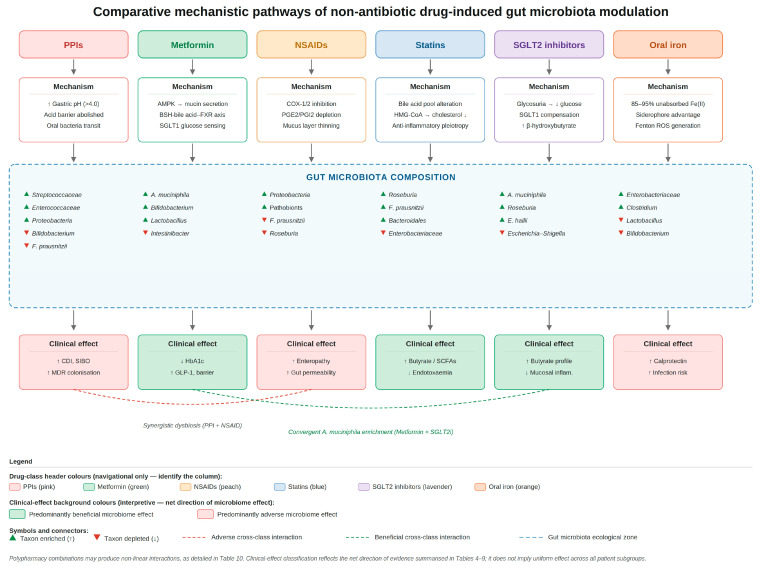
Comparative overview of the mechanistic pathways through which PPIs, metformin, NSAIDs, statins, SGLT2 inhibitors, and oral iron supplements modulate gut microbiota composition. Each drug-class column summarises, from top to bottom, the principal molecular mechanism of microbiome perturbation, the directionality of compositional change in representative taxa (▲ enrichment, ▼ depletion), and the dominant clinical consequence relevant to pharmacotherapy and pharmacovigilance. The dashed border around the central panel delineates the gut microbiota ecological zone in which these effects converge. Colour coding within the figure is defined in the legend at the foot of the panel and operates at two distinct levels. The six coloured boxes at the top of each column (PPIs, pink; metformin, green; NSAIDs, peach; statins, blue; SGLT2 inhibitors, lavender; oral iron, orange) serve a navigational function only, identifying each drug-class column. By contrast, the shaded background of the “Clinical effect” boxes at the bottom of each column carries interpretive meaning: light green denotes a predominantly beneficial microbiome effect (metformin, statins, SGLT2 inhibitors), whereas light pink denotes a predominantly adverse microbiome effect (PPIs, NSAIDs, oral iron). This dichotomous classification reflects the net direction of the available pharmacomicrobiomic evidence summarised in Table 4, Table 5, Table 6, Table 7, Table 8 and Table 9 and does not imply a uniform effect across all patient subgroups. PPIs abrogate the gastric acid barrier, permitting oralisation of the gut (↑ Streptococcaceae, Enterococcaceae, Proteobacteria; ↓ Bifidobacterium, *F. prausnitzii*) with clinical consequences including increased risk of CDI, SIBO and multidrug-resistant organism colonisation. Metformin drives beneficial remodelling via *Akkermansia muciniphila* expansion and bile acid–FXR axis modulation (↑ *A. muciniphila*, Bifidobacterium, Lactobacillus; ↓ Intestinibacter), supporting glycaemic control and barrier integrity. NSAIDs deplete anti-inflammatory commensals through COX-dependent prostaglandin depletion (↑ Proteobacteria and pathobionts; ↓ *F. prausnitzii*, Roseburia), increasing enteropathy and gut permeability. Statins enrich SCFA-producing taxa through bile acid pool alteration (↑ Roseburia, *F. prausnitzii*, Bacteroidales; ↓ Enterobacteriaceae), with downstream increases in butyrate/SCFA and reduced endotoxaemia. SGLT2 inhibitors promote butyrate-producing genera through glycaemia-mediated mucosal effects (↑ *A. muciniphila*, Roseburia, E. hallii; ↓ Escherichia–Shigella), improving the mucosal butyrate profile and reducing inflammation. Oral iron supplements favour siderophilic Enterobacteriaceae at the expense of Lactobacillus and Bifidobacterium (↑ Enterobacteriaceae, Clostridium; ↓ Lactobacillus, Bifidobacterium), increasing faecal calprotectin and infection risk. Dashed connectors at the base of the figure highlight two clinically important cross-class interactions emphasised in the text: synergistic dysbiosis under PPI + NSAID co-prescription (red dashed connector), and convergent *A. muciniphila* enrichment under metformin + SGLT2i combination therapy (green dashed connector). The figure is intended as a comparative schematic rather than a quantitative depiction of effect magnitude, which varies substantially across drug classes (highest confidence for PPIs and metformin; lowest for SGLT2 inhibitors and oral iron in adults), as detailed in Section 2.2 and Section 3.

Oral iron co-administered with PPIs represents a common clinical scenario that may compound dysbiosis: PPI-induced loss of acid-mediated colonisation resistance combined with iron-driven siderophilic enrichment of *Enterobacteriaceae* creates overlapping selection pressures favouring Gram-negative pathobionts. Conversely, the Statin + Oral iron combination in anaemic cardiovascular patients could represent a partially balanced scenario, with statin-driven anti-inflammatory microbiome effects potentially attenuating iron-induced mucosal inflammation. Table 10 presents mechanistic hypotheses and available evidence for key polypharmacy scenarios. Figure 2 provides a comparative overview of the mechanistic pathways by which all six drug classes modulate gut microbiota.

## 4. Discussion

### 4.1. Principal Findings and Pharmacological Significance

This narrative review synthesises evidence from 68 studies (20 clinical studies tabulated and 48 preclinical and prior systematic reviews integrated narratively) and demonstrates, with qualitatively higher confidence for established drug classes (PPIs, metformin) and lower confidence for emerging classes (SGLT2 inhibitors, oral iron), that six widely prescribed non-antibiotic drug and supplement classes produce mechanistically distinct, clinically significant, and frequently underrecognised effects on the human gut microbiota. The convergence of findings across large-scale population cohorts, placebo-controlled RCTs, and germ-free experimental models with human validation confers substantial confidence in the biological reality of these pharmacomicrobiome interactions. Six overarching conclusions emerge with high consistency across the evidence base, each accompanied below by an explicit indication of evidence strength to support symmetric comparison.

First (evidence strength: high, based on large multi-cohort observational studies with consistent directionality and partial twin-control replication; limited by predominance of cross-sectional designs), PPIs are associated with the most consistently adverse microbiome profile among the six drug classes. The enrichment of oral-origin taxa in the colon during PPI therapy is best interpreted as a fundamental disruption of the ecological zonation that characterises a healthy gastrointestinal tract. The magnitude of this effect in population studies equals or exceeds that of major dietary and lifestyle determinants [3,5,88], underscoring that this widely used drug class produces pharmacological consequences far beyond its intended target organ. Of particular clinical concern is the persistence of dysbiosis documented at 6 months post-initiation [51], which suggests, though does not formally establish, that patients who discontinue PPIs after prolonged use remain at elevated risk of dysbiosis-related complications, CDI, SIBO, and multidrug-resistant colonisation, for a period substantially outlasting the pharmacological exposure.

Second (evidence strength: high; the only drug class in this review with formal Hill-criteria-level causal evidence from germ-free colonisation and FMT experiments combined with adequately powered human RCTs), metformin provides one of the most compelling pharmacomicrobiome paradigms in clinical medicine: a drug whose therapeutic efficacy is plausibly partly mediated through, rather than despite, its gut microbiome interactions. The causal chain from drug exposure to *A. muciniphila* enrichment, improved mucosal barrier integrity, FXR-mediated suppression of hepatic gluconeogenesis, and glycaemic control is now supported by convergent RCT, FMT, and metabolomics evidence [22,40,64,105], with *A. muciniphila* increasingly recognised as a next-generation probiotic candidate whose metabolic benefits extend across the gut–liver–brain axis [106]; the relative contribution of microbiota-mediated versus hepatic mechanisms to overall glycaemic efficacy nevertheless remains an open quantitative question.

Third (evidence strength: intermediate; based on a small number of human observational cohorts, one germ-free mouse causal study and converging mechanistic data, with limited adequately powered RCTs), the pharmacological paradox of NSAIDs, depleting the microbiome’s key endogenous anti-inflammatory regulator (*F. prausnitzii*) while inhibiting eicosanoid-mediated inflammation, has direct therapeutic implications. The demonstration that PPI co-prescription synergistically amplifies rather than mitigates small intestinal microbiome damage [76] provides a strong rationale to reassess the standard gastroprotective rationale for PPI + NSAID co-prescription, at least for prolonged exposure and in patients at elevated mucosal risk.

Fourth (evidence strength: intermediate, dominated by one large population cohort and several preclinical studies, with few interventional human data), statins emerge as a drug class with predominantly favourable pharmacomicrobiome interactions. The enrichment of butyrate-producing taxa and partial correction of obesity-linked dysbiosis documented in population-level studies [25,85] suggest that a proportion of statins’ cardiovascular and metabolic benefits may be microbiome-mediated, analogous to the metformin paradigm but operating through bile acid-mediated rather than mucin-mediated mechanisms; this analogy is heuristic and should not be taken to imply equivalent maturity of clinical evidence.

Fifth (evidence strength: low for SGLT2 inhibitors in adults, given the limited and discordant RCT data; intermediate for oral iron, anchored by adequately powered paediatric RCTs but with limited adult evidence), the emerging evidence on SGLT2 inhibitors and oral iron supplements completes a pharmacomicrobiome spectrum ranging from clearly beneficial (metformin, statins) through mixed (SGLT2i) to clearly adverse (PPIs, oral iron). The nutritional relevance of oral iron-induced dysbiosis is particularly salient for this journal’s scope: the demonstration that standard iron supplementation protocols systematically enrich siderophilic pathobionts at the expense of beneficial commensals [30,44] has direct implications for iron fortification policy, antenatal supplementation guidance, and the design of next-generation iron formulations with reduced microbiome disruption.

Sixth (evidence strength: methodological rather than clinical, reflecting the integration of preclinical and prior systematic reviews into the synthesis), the mechanistic depth of the present synthesis owes substantially to the 48 preclinical and prior systematic review studies that were not tabulated as primary clinical evidence but were integrated within the narrative sections for each drug class (Figure 1). These supporting investigations, encompassing germ-free and gnotobiotic murine models, faecal microbiota transplantation experiments, in vitro anaerobic culture systems, and high-throughput drug–microbiota screening platforms, were indispensable for establishing causal directionality between drug exposure and microbial perturbation, a determination that observational clinical data alone cannot provide. For metformin, germ-free mouse colonisation and FMT experiments [22,40] satisfied modified Hill criteria for causality by demonstrating that drug-induced microbial shifts are both necessary and sufficient for the observed metabolic phenotype. For NSAIDs, germ-free models established that microbiota composition critically modulates indomethacin-induced enteropathy severity and that FMT from healthy donors attenuates small intestinal injury [41]. For oral iron, in vitro siderophore competition assays and human gut microbiota-associated rat models [95,99] elucidated the molecular basis of *Enterobacteriaceae* competitive advantage under high-iron conditions. More broadly, the landmark in vitro screening by Maier et al. (2018), which demonstrated that 24% of non-antibiotic drugs inhibit at least one gut commensal strain [19], together with the bioaccumulation paradigm described by Klünemann et al. (2021) showing that gut bacteria can sequester therapeutic drugs intracellularly without chemical modification [107], and the systematic mapping of microbial drug-metabolising genes by Zimmermann et al. (2019) [10], have collectively transformed pharmacomicrobiomics from a correlational discipline into a mechanistically grounded field. Recent comprehensive reviews by Weersma et al. (2020) [108] and Zimmermann et al. (2021) [109] have further systematised the bidirectional framework of drug–microbiome interactions, while Javdan et al. (2020) demonstrated personalised mapping of drug metabolism by the human gut microbiome using individual-level ex vivo culture systems [110], and the large-scale environmental covariate analysis by Gacesa et al. (2022) confirmed that medication use ranks among the most influential exogenous determinants of gut microbiome composition in the general population [111]. The integration of these preclinical and methodological advances with the 20 tabulated clinical studies provides a translational evidence architecture in which clinical associations are anchored by experimentally validated causal mechanisms, a standard of evidence increasingly demanded by regulatory pharmacovigilance frameworks and essential for the clinical implementation of microbiome-informed prescribing strategies [112].

In the context of non-antibiotic drug effects on antimicrobial resistance, three distinct categories of evidence should be carefully separated and not conflated. First, in vitro non-antibiotic antimicrobial activity findings, most notably the Maier et al. screen demonstrating that ~24% of marketed non-antibiotic drugs inhibit at least one gut commensal at clinically relevant concentrations [19], establish microbial vulnerability to these drugs under controlled conditions but do not directly demonstrate selection of resistance in vivo. Second, observational resistome associations, such as the increased relative abundance of tetracycline- and macrolide-efflux resistance genes (tetA, tetB, Mel) in PPI users reported by Vich Vila et al. [5], identify gene-level correlates of drug exposure but cannot, by themselves, establish that these gene-level shifts translate into clinically meaningful resistant infection. Third, clinically validated resistance outcomes, such as the meta-analytic association between PPI use and intestinal colonisation by multidrug-resistant organisms [53], are the most directly actionable but remain comparatively rare in the pharmacomicrobiomic literature for non-antibiotic drugs. Throughout this review, statements about non-antibiotic-drug contributions to the resistome are framed at the level of evidence available (in vitro, observational gene-level, or clinical outcome), and are not extrapolated across these tiers without explicit justification.

### 4.2. Quality Assessment and Evidence Grading

Methodological quality of included clinical studies was qualitatively appraised by the authors based on study design (with randomised controlled trials and prospective cohorts considered higher quality than cross-sectional analyses), sample size, control group adequacy, and microbiome characterisation methodology (with shotgun metagenomics and standardised 16S rRNA pipelines considered higher quality than non-standardised approaches). Formal scoring instruments (Newcastle–Ottawa Scale [113], Cochrane RoB 2 [114], GRADE [115]) were not applied, consistent with the narrative review design.

### 4.3. Clinical and Pharmaceutical Implications

**Figure 3:**
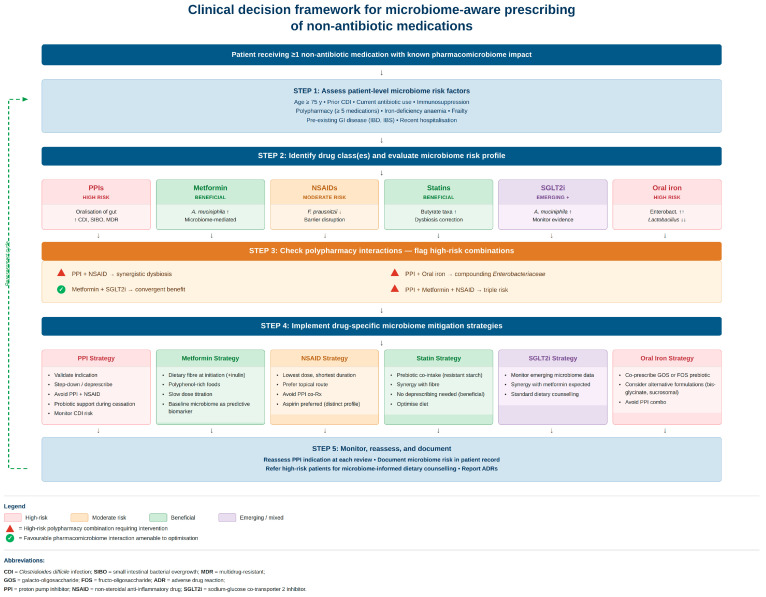
Proposed clinical decision framework for microbiome-aware prescribing of six non-antibiotic drug classes. The algorithm integrates patient-level risk factors (immunosuppression, prior CDI, polypharmacy status, age ≥ 75 years, iron-deficiency anaemia, cardiovascular comorbidity) with drug-specific microbiome risk profiles and evidence-based mitigation strategies (structured deprescribing, dietary fibre co-supplementation, probiotic adjuncts, prebiotic co-administration, alternative iron formulations). Colour coding within the figure is defined in the legend at the foot of the panel and operates at the box level (not at the level of arrows or connectors). Pink-shaded boxes denote drug classes and mitigation strategies classified as high-risk (PPIs, oral iron), peach-shaded boxes denote moderate-risk classes (NSAIDs), light-green-shaded boxes denote classes with beneficial pharmacomicrobiome interactions amenable to optimisation (metformin, statins), and lavender-shaded boxes denote classes with emerging or mixed evidence pending prospective validation (SGLT2 inhibitors). Within STEP 3, a red triangle (▲) flags a high-risk polypharmacy combination requiring active pharmacist intervention, whereas a green checkmark identifies a favourable polypharmacy interaction amenable to optimisation. The green dashed connector running along the left margin from STEP 5 to STEP 1 represents the reassessment cycle. Risk-level classifications reflect the net direction of the pharmacomicrobiomic evidence summarised in Table 4, Table 5, Table 6, Table 7, Table 8 and Table 9 and Section 3.3, Section 3.4, Section 3.5, Section 3.6, Section 3.7 and Section 3.8 and are intended to support, not replace, individualised clinical judgement.

The recommendations developed in this section vary in the evidence base that supports them, and this hierarchy should be made explicit before they are considered for clinical adoption, consistent with generally accepted principles for grading the strength of clinical recommendations [115,116]. Two recommendations are the most directly supported by the evidence reviewed and may reasonably be considered evidence-supported: (i) structured PPI deprescribing in patients without a continuing indication, particularly those with additional microbiome-related risk factors; and (ii) prebiotic co-administration (e.g., GOS) with oral iron in vulnerable populations, where the most direct RCT evidence exists. By contrast, several other recommendations, including metformin–polyphenol or fibre synergy, prebiotic co-intake with statins, and microbiome-rationalised combination strategies for SGLT2 inhibitors, are currently better characterised as speculative optimisation strategies extrapolated from mechanistic and observational data, pending prospective clinical validation [115]. The following paragraphs are written with this hierarchy in mind, and Section 3.3 should be read together with Figure 3 as a framework for clinical reasoning rather than as a balanced set of prescribing rules.

For PPIs, the evidence supports structured deprescribing protocols incorporating microbiome-related risk stratification. Medication review processes should be implemented to assess PPI indication validity, attempt step-down therapy, and identify patients at compound microbiome risk: those simultaneously receiving NSAIDs, who have prior CDI, whose mortality risk is compounded by advanced age, comorbidity burden, and polypharmacy as demonstrated in Eastern European cohort data [52], or antibiotic exposure, who are of advanced age (≥75 years), or who are immunocompromised by disease or therapy [6,53]. The duration-dependent SIBO risk documented by Lombardo et al. (2010) [51] suggests that enhanced vigilance and potential probiotic or prebiotic support during the months following PPI cessation may be warranted in high-risk patients [117,118]. However, the evidence for probiotic prophylaxis against PPI-associated CDI remains inconsistent: while Cochrane meta-analytic data support modest protective efficacy of selected probiotic strains against antibiotic-associated CDI [119], recent evidence demonstrates that empiric probiotic administration may paradoxically delay post-perturbation mucosal microbiome reconstitution compared with autologous FMT [120], and that probiotic colonisation efficacy is highly individualised and dependent on host-specific mucosal features [121], underscoring the need for personalised rather than empiric approaches to microbiome restoration in PPI-deprescribed patients.

For metformin, dietary co-interventions at initiation represent a mechanistically plausible but as of yet incompletely validated optimisation strategy and should be formally incorporated into prescribing guidance only as adequately powered prospective evidence accumulates. Fibre supplementation, polyphenol-rich functional foods, and fermented beverages provide accessible and biologically plausible complements to formal drug therapy, potentially expanding the *A. muciniphila*-enriching effects of metformin and attenuating GI adverse effects associated with *Escherichia* expansion [31,40,66]. The prospective validation of baseline *A. muciniphila* abundance as a predictive biomarker for metformin response, and *Escherichia* as a predictor of GI intolerance, represents a pharmacomicrobiome precision medicine priority that could transform metformin prescribing from a population-level approach to a microbiome-stratified strategy.

For NSAIDs, the evidence supports restriction to the lowest effective dose for the shortest necessary duration, preference for topical or short-acting formulations where clinically feasible, and critical reassessment of PPI co-prescription policies in light of evidence for compounded small intestinal dysbiosis [76,77]. The distinct microbiome profile of aspirin versus non-selective NSAIDs [73] warrants investigation as a prescribing differentiator.

For statins, the predominantly favourable microbiome profile provides additional mechanistic rationale for guideline-recommended statin therapy in cardiovascular prevention. Prebiotic dietary co-interventions (inulin, resistant starch) constitute a speculative optimisation hypothesis that may synergistically amplify statin-driven enrichment of butyrate-producing taxa, representing a pharmaconutritional optimisation strategy warranting prospective evaluation [25,85].

For SGLT2 inhibitors, the emerging evidence of favourable microbiome remodelling adds a mechanistic dimension to their established cardiorenal benefits. The convergent enrichment of *A. muciniphila* by both metformin and SGLT2i [28,91] suggests that their combination, increasingly standard in T2DM management, may produce synergistic microbiome benefits deserving dedicated investigation; this remains an explicitly speculative hypothesis at present.

For oral iron supplements, the evidence most directly supports co-administration of prebiotics (GOS, FOS) to mitigate iron-induced dysbiosis [44]—the strongest evidence-based recommendation in this list, anchored by adequately powered paediatric RCTs, a strategy of particular relevance in anaemic populations with pre-existing microbiome vulnerability (infants, pregnant women, elderly). Alternative iron formulations with reduced colonic iron delivery, including sucrosomial iron, iron bisglycinate, and slow-release preparations, warrant microbiome-focused comparative evaluation as potential dysbiosis-mitigating strategies [29]. A proposed clinical decision framework integrating these risk factors is presented in Figure 3.

## 5. Conclusions

This narrative review synthesises 68 studies, comprising 20 clinical studies in human participants (tabulated as the core evidence base) and 48 preclinical mechanistic investigations and prior systematic reviews integrated narratively, and shows that six widely prescribed non-antibiotic drug classes (PPIs, metformin, NSAIDs, statins, SGLT2 inhibitors, and oral iron) exert distinct and clinically significant effects on the gut microbiota, with direct clinical and nutritional implications.

PPIs produce the most adverse profile, reducing diversity and enriching oral-origin taxa, with dysbiosis persisting beyond discontinuation and raising risks of CDI, SIBO, and MDR colonisation. Metformin drives predominantly favourable remodelling via *A. muciniphila* expansion and FXR–bile acid modulation, with baseline microbiome composition predicting efficacy and GI tolerability. NSAIDs deplete anti-inflammatory commensals (notably *F. prausnitzii*) and compound mucosal injury when combined with PPIs. Statins and SGLT2 inhibitors show favourable profiles, enriching butyrate producers and *A. muciniphila* through bile acid and glycaemia-mediated mechanisms that plausibly contribute to their cardiovascular and cardiorenal benefits. Oral iron supplements, in contrast, systematically enrich siderophilic *Enterobacteriaceae* at the expense of *Lactobacillus* and *Bifidobacterium*, with prebiotic co-supplementation as an evidence-based mitigation.

Collectively, these findings position pharmacomicrobiomics as a clinically actionable discipline. Key practice implications include structured PPI deprescribing, dietary fibre and polyphenol co-prescription with metformin, reassessment of PPI + NSAID combinations, and routine prebiotic co-administration with oral iron in vulnerable populations. Forward-looking applications such as microbiome-active drug delivery systems (MADDS) further extend the translational reach of pharmacomicrobiomic data beyond prescribing into formulation design. Future work should prioritise prospective RCTs integrating metagenomics and clinical outcomes, validation of microbiome-based biomarkers for drug response, and dedicated studies in underrepresented populations.

## Figures and Tables

**Table 1:** PICO framework defining the eligibility scope of the systematic search.

PICO	Specification
P—Population	Adult humans ≥ 18 years receiving non-antibiotic pharmacological therapy; no restriction on sex, ethnicity, or comorbidity
I—Intervention	Exposure to ≥1 target drug class: proton pump inhibitors (PPIs), metformin, NSAIDs, statins (HMG-CoA reductase inhibitors), SGLT2 inhibitors, or oral iron supplements; any dose, route, or duration
C—Comparison	Drug-naive controls, placebo arms in RCTs, or within-subject pre-treatment baselines
O—Primary	Changes in alpha diversity (Shannon, richness, Faith’s PD), beta diversity (UniFrac, Bray–Curtis), and differential taxon abundance
O—Secondary	CDI and SIBO incidence; glycaemic/metabolic parameters; lipid profiles; intestinal permeability markers; iron status markers; GI adverse events

**Table 2:** Inclusion and exclusion criteria applied during systematic study selection.

Inclusion Criteria	Exclusion Criteria
Human clinical studies (RCT, cohort, cross-sectional, case–control)	Animal-only or exclusively in vitro/ex vivo studies
≥1 target drug class (PPI, metformin, NSAIDs, statins, SGLT2i, oral iron) as primary or adjunct intervention	Antibiotics as the primary intervention of interest
Gut microbiota characterised by 16S rRNA sequencing, shotgun metagenomics, or validated qPCR	Conference abstracts, letters, editorials, case reports (n < 5)
Published January 2015–December 2024; English language	Sample size < 20 participants
Sample size ≥ 20; ≥4-week drug exposure or adequate crossover washout	Duplicate publications; secondary subgroup analyses
Available full text; ≥1 primary outcome extractable	Narrative reviews without primary data

**Table 3:** Pharmacomicrobiomic evidence confidence grading by drug class.

Drug Class	Clinical Studies (*n*)	Predominant Study Designs	Supporting Mechanistic Evidence (Preclinical)	Confidence	Translational Readiness
Metformin	5	RCTs and prospective cohorts	Strong: germ-free murine models, faecal microbiota transplantation, gnotobiotic colonisation studies	High	Established
Proton pump inhibitors (PPIs)	5	Large observational cohorts (cross-sectional and longitudinal)	Moderate: in vitro acid suppression models and animal microbiota studies	High	Established
Statins	3	RCT and observational cohorts	Limited: in vitro antimicrobial assays; sparse animal data	Intermediate	Emerging
NSAIDs	2	Observational cohorts	Moderate: animal models of NSAID enteropathy and barrier dysfunction	Intermediate	Emerging
Oral iron	3	RCTs (paediatric) and cohorts	Moderate: animal models, in vitro siderophilic pathobiont enrichment	Low	Hypothesis-generating
SGLT2 inhibitors	2	RCT and observational cohort	Limited: sparse preclinical data	Low	Hypothesis-generating

Abbreviations: RCT, randomised controlled trial; NSAID, non-steroidal anti-inflammatory drug; PPI, proton pump inhibitor; SGLT2, sodium-glucose co-transporter 2.

**Table 4:** Key clinical studies evaluating proton pump inhibitor (PPI) effects on gastrointestinal microbiota composition, diversity, and clinical outcomes (chronological order).

Author, Year	Design	N	Agent & Duration	Key Microbiota Finding	Pharmacological/Clinical Consequence
Freedberg et al., 2015 [34]	Open-label crossover interventional trial	12 healthy volunteers	Omeprazole 40 mg twice daily, 4 weeks	No change in α-diversity (Shannon *p* = 0.71); ↑ *Enterococcaceae* (*p* = 0.03), *Streptococcaceae* (>10-fold, *p* < 0.01), *Micrococcaceae* (*p* = 0.01), *Staphylococcaceae* (*p* = 0.01); ↓ *Clostridiaceae* (44%, *p* = 0.03). No change in faecal bile acids; predicted ↑ bacterial invasion pathways (PICRUSt).	Causal evidence (independent of antibiotic confounding) that PPI exposure shifts microbiota toward CDI-permissive composition via impaired colonisation resistance not via altered bile acid metabolism—a clean pharmacomicrobiomic mechanism for the well-documented PPI–CDI ADR.
Imhann et al., 2016 [3]	Cross-sectional, three-cohort meta-analysis	1815 (1174 general population + 300 IBD + 341 IBS case–control)	Multiple PPIs (omeprazole, esomeprazole, pantoprazole, lansoprazole, dexlansoprazole, rabeprazole)	92/460 taxa significantly altered (~20%; FDR < 0.05). ↑ oral-origin genera (*Rothia*, *p* = 9.8 × 10^−38^; *Streptococcus*, *Veillonella*). ↑ potential pathogens (*Enterococcus*, *Staphylococcus*, *E. coli*). ↓ Shannon diversity; significant shift toward oral microbiome composition (PCoA1 *p* = 1.39 × 10^−20^).	Largest population-level pharmacomicrobiomic evidence of PPI-induced “oralisation of the gut”. Taxonomic shifts overlap with *C. difficile* susceptibility signatures, providing mechanistic context for elevated enteric infection risk in PPI users.
Jackson et al., 2016 [4]	Cross-sectional twin cohort with discordant MZ-pair paired analysis	1827 (TwinsUK; 70 MZ-discordant pairs)	Multiple PPIs (self-reported, not stratified)	↑ oral-origin taxa (*Streptococcaceae* q < 10^−6^; *Micrococcaceae* q < 10^−5^; *Rothia mucilaginosa*, *Streptococcus anginosus*). ↓ gut commensals *Lachnospiraceae* and *Ruminococcaceae*. α-diversity reduction lost significance after covariate adjustment; effects confirmed in MZ-discordant pairs (genetic-independent) and replicated in interventional data.	Genetic-independent confirmation of PPI-induced “oralisation”, strengthening causal inference under modified Hill criteria. Depletion of butyrogenic commensals and enrichment of oral-type taxa parallel signatures associated with *C. difficile* susceptibility.
Hojo et al., 2018 [37]	Prospective pre–post study (RT-qPCR)	20 patients with reflux oesophagitis	Esomeprazole 20 mg (n = 16), rabeprazole 10 mg (n = 2), lansoprazole 30 mg (n = 2); 8 weeks	↑ *Lactobacillus* spp. (incl. *L. gasseri*, *L. fermentum*, *L. reuteri*, *L. ruminis* subgroups). ↑ *Streptococcus*; ↑ *Enterobacteriaceae* and *Staphylococcus* at 8 weeks. No significant change in obligate anaerobes, total organic acids, or faecal pH; transient ↑ formic and butyric acid at 4 weeks.	Quantitative confirmation (RT-qPCR) of dysbiosis previously described by metagenomics. Oral-type *Streptococcus* (*S. salivarius*, *S. oralis*) detected in blood post-treatment, suggesting, but not proving, bacterial translocation of pharmacovigilance relevance.
Shi et al., 2019 [42]	Cross-sectional observational (16S rRNA, V4)	55 faecal (15 HC, 15 non-PPI, 25 PPI); 35 gastric mucosa (5 HC, 10 non-PPI, 20 PPI)	Omeprazole 40 mg/day; short-term (<1 yr) vs. long-term (>1 yr) stratification	Gastric mucosa: ↓ richness in PPI users (Chao1, observed species); ↑ *Planococcaceae*, *Oxalobacteraceae*, *Sphingomonadaceae*; ↑ *Methylophilus* in long-term users. Faecal: no change in α-diversity; ↑ *Streptococcaceae*, *Veillonellaceae*, *Acidaminococcaceae*, *Micrococcaceae*, *Flavobacteriaceae* in PPI users; ↑ *Ruminococcus* in long-term users.	PPI therapy compartment specifically alters gastric mucosal community structure and diversity, with milder, composition-only effects on faecal microbiota. Duration-dependent shifts suggest progressive pharmacomicrobiomic remodelling with sustained exposure relevant to long-term PPI deprescribing decisions.

Abbreviations and symbols: ↑, increase/enrichment relative to comparator or baseline; ↓, decrease/depletion relative to comparator or baseline.

**Table 6:** Clinical evidence for NSAID-induced alterations in gut microbiota composition.

Author, Year	Design	N	Key Microbiota Finding	Principal Taxa Affected	GI Outcome
Rogers & Aronoff, 2016 [23]	Cross-sectional observational study Adult community cohort, Michigan, USA 16S rRNA sequencing (V3–V5 region) on 454 pyrosequencing platform OTU analysis with mothur + RDP Bayesian classifier Logit models with AUC for discriminating microbial profiles	155 adults (mean age 52 years) 62 NSAID users (40%) 16.8% with no medication in the previous 30 days	Drug type influenced microbiome more than number of drugs taken NSAID-associated microbial profiles were molecule-specific Aspirin: discriminated by four OTUs (*Prevotella*, *Bacteroides*, Ruminococcaceae, *Barnesiella*); AUC = 0.96 Ibuprofen ≈ celecoxib (similar profiles): ↑ Acidaminococcaceae and Enterobacteriaceae Ibuprofen vs. naproxen: ↑ Propionibacteriaceae, Pseudomonadaceae, Puniceicoccaceae, Rikenellaceae in ibuprofen users Naproxen: discriminated by Enterobacteriaceae, three *Bacteroides* spp., *Alistipes* spp.; AUC = 0.92	↑ Enterobacteriaceae (ibuprofen, celecoxib, naproxen users) ↑ Acidaminococcaceae (ibuprofen, celecoxib) Marked variation in *Prevotella*, *Bacteroides*, Ruminococcaceae ↑ Desulfovibrionaceae and Enterococcaceae across NSAID users Ibuprofen: ↑ *Prevotella* spp., *Alistipes* spp. Naproxen: ↑ specific *Bacteroides* spp.	Drug-specific microbiome signatures shaped by NSAID type and co-medication NSAID + PPI combinations alter microbiota distinctly, suggesting exacerbation of distal enteropathy Possible link to choline metabolism → TMA/TMAO and differential cardiovascular risk between ibuprofen and naproxen Microbiota reflects the full drug combination, not isolated NSAID exposure
Vich Vila et al., 2020 [5]	Multi-cohort meta-analysis of three independent Dutch cohorts: LifeLines-DEEP (general population), 1000IBD (UMCG), Maastricht IBS case–control Shotgun metagenomic sequencing (Illumina HiSeq, Broad Institute) Profiling with MetaPhlAn2 + HUMAnN2 + ShortBRED (resistome) Single-drug and multi-drug analyses adjusted for age, sex, BMI, sequencing depth, disease	1883 total participants 1124 LifeLines-DEEP + 454 IBD + 305 IBS 85 NSAID users in total (42 + 21 + 22) 41 drug categories analysed; 537 distinct drug combinations identified	Single-drug analysis: 19 of 41 drug categories showed significant microbiota associations Multi-drug analysis (correcting for polypharmacy): only four drugs retained dominant impact—PPIs, metformin, antibiotics, laxatives NSAIDs did NOT remain among dominant-impact drugs after polypharmacy correction Number of concomitant drugs correlated with global compositional changes (PERMANOVA, FDR < 0.05 in all cohorts)	PPIs: most consistent signature—↑ *Streptococcus salivarius*, *S. parasanguinis*, *S. vestibularis*, *Veillonella parvula*, *Bifidobacterium dentium* Metformin: ↑ *Escherichia coli*, ↓ *Intestinibacter bartlettii* Broad drug-dependent taxonomic and functional changes PPIs: 24 taxa + 133 metabolic pathways altered (after multi-drug) Metformin: 48 metabolic pathways altered (no significant taxa after multi-drug) Laxatives: ↑ *Alistipes*, *Bacteroides* spp. Antibiotics: ↓ *Bifidobacterium* ↑ resistance genes *tetA*, *tetB*, *Mel* (tetracycline/macrolide efflux) in PPI users across all cohorts	Polypharmacy is a major confounder in microbiome studies, adjustment for concomitant drug use is essential Patients with GI disease (IBD, IBS) show distinct polypharmacy patterns that affect interpretation Pharmacomicrobiomics must integrate clinical context (multi-medication + comorbidities) Implications for antimicrobial resistance: non-antibiotic drugs contribute to the resistome Foundation for future investigations of microbiota-mediated adverse effects

Abbreviations and symbols: ↑, increase/enrichment relative to comparator or baseline; ↓, decrease/depletion relative to comparator or baseline.

**Table 7:** Key clinical studies evaluating statin effects on gut microbiota composition and metabolic–cardiovascular outcomes.

Author, Year	Study Design	N	Key Microbiota Findings	Principal Taxa Affected	Clinical Implication
Vieira-Silva et al., 2020 [25]	Cross-sectional MetaCardis study; quantitative shotgun metagenomic profiling (QMP) Microbial-load–normalised pipeline; Dirichlet-multinomial enterotyping Discovery: BMIS cohort; validation: MetaCardis CVD + Flemish Gut Flora Project (FGFP) Stepwise dbRDA covariate analysis adjusting for obesity, metabolic syndrome and co-medications	888 BMIS (106 statin users; simvastatin 48%, atorvastatin 31%) Validation: 282 CVD + 2345 FGFP BMI range 18.0–73.3 kg/m^2^	Statin therapy: strongest medication covariate of genus-level microbiome variation (R^2^ = 0.24%, P_adj_ = 0.032) Bact2 enterotype (high *Bacteroides*, low *Faecalibacterium*, low microbial load) associated with systemic inflammation and obesity Bact2 prevalence in obese: 17.73% (non-statin) vs. 5.88% (statin); Fisher *p* = 0.028 Replicated in CVD (16.33% vs. 4.72%, *p* = 0.008) and FGFP (RR = 0.72, P_adj_ = 0.045) Statin effect on Bact2 reduction partly independent of hsCRP and HbA1c (multivariate RR = 0.36, P_adj_ = 0.039)	Bact2 enterotype depleted in statin users *Akkermansia* (metabolic-health-associated) preserved *Faecalibacterium* (anti-inflammatory) preserved ↓ *Bacteroides fragilis* (Bact2-enriched) ↓ *Eggerthella* (Bact2-enriched, infection-linked)	First large-scale evidence that statin therapy is associated with reduced microbiota dysbiosis in obesity Effect extends beyond anti-inflammatory action, suggesting direct/indirect microbiota modulation Statins proposed as candidate microbiota-modulating therapeutics (prospective RCT validation needed) Reframes statins as having pleiotropic gut ecosystem benefits relevant to obesity comorbidities
Hu et al., 2021 [39]	Prospective single-centre cohort (PUMCH); median 2.16-year follow-up 16S rRNA sequencing (V3–V4) + untargeted serum metabolomics (UPLC-Q-TOF-MS) PICRUSt2 functional pathway prediction (MetaCyc) In vitro validation: anaerobic culture with atorvastatin/rosuvastatin; SCFA by GC-MS	133 subjects: 36 ACS on chronic statin + 67 ACS statin-naïve + 30 controls Follow-up: 68 ACS patients (26 statin, 42 non-statin)	Statins shifted ACS gut microbiome towards a healthier intermediate state between ACS and controls ↑ *Bifidobacterium longum* subsp. *longum*, *Anaerostipes hadrus*, *Ruminococcus* (*Blautia*) *obeum* ↓ *Parabacteroides merdae* and overall potentially pathogenic taxa (BugBase) Multi-omics: *P. merdae* linked to adverse outcomes via prenol lipid mediator cyclopassifloside II; *R. obeum* linked to favourable outcomes via nervonic acid, docosanamide PICRUSt2: ↑ methanogenesis from acetate, pyrimidine and amino-acid pathways; ↓ menaquinol-8 biosynthesis II	↑ *B. longum* subsp. *longum* (probiotic) ↑ *A. hadrus* (butyrate producer; in vitro butyrate ↑ with atorvastatin) ↑ *R.* (*Blautia*) *obeum* (propionate producer) ↓ *P. merdae* (in vitro growth inhibited by atorvastatin) ↓ *Bacteroides* (downward trend, consistent with Vieira–Silva)	Chronic prior statin therapy associated with improved ACS outcomes (composite endpoint OR = 0.10, *p* = 0.030) Cardiovascular benefit partly mediated through gut microbiota–metabolite remodelling (SCFA, prenol lipid, fatty acid axes) In vitro mechanistic confirmation of direct statin–microbe interactions Supports microbiota as a co-target underlying statin pleiotropy in ACS
Zhernakova et al., 2016 [88]	Population-based metagenomic shotgun sequencing (LifeLines-DEEP, Netherlands) MetaPhlAn 2.0 taxonomic profiling; HUMAnN/MetaCyc functional profiling Multivariate association of 207 intrinsic and exogenous factors with microbiome composition, diversity and gene/COG richness	1135 participants from the Dutch general population 56 statin users among 44 drug categories analysed	Statin use among the medication classes most strongly associated with gut microbiota composition (FDR < 0.1) Statins contributed independent explanatory power to inter-individual Bray–Curtis variation Microbiota signatures of statin users overlapped partially with those of PPIs and metformin, highlighting polypharmacy as a key confounder Findings consistent with subsequent evidence that statins shift composition away from dysbiotic configurations	Statin use associated with shifts in multiple genus-level taxa (composition-level effect; no single dominant taxon) Overlap with calprotectin- and PPI-associated oral-origin bacteria (e.g., *Streptococcus*, *Veillonella*) Supports preserved diversity in statin users at population scale	Independent population-level confirmation that statins are non-trivial modulators of the gut microbiome outside disease cohorts Establishes general-population baseline against which clinical statin–microbiota effects can be interpreted Reinforces need to adjust for statin use in any microbiome–cardiometabolic association study Methodological framework later adopted by MetaCardis (Vieira-Silva 2020 [25])

Abbreviations and symbols: ↑, increase/enrichment relative to comparator or baseline; ↓, decrease/depletion relative to comparator or baseline.

**Table 8:** Key clinical studies evaluating SGLT2 inhibitor effects on gut microbiota composition and cardiometabolic outcomes.

Author, Year	Study Design	N	Key Microbiota Findings	Principal Taxa Affected	Clinical Implication
van Bommel et al., 2020 [92]	Double-blind, comparator-controlled, parallel-group RCT (12 weeks) Add-on to stable metformin therapy 16S rRNA sequencing (V3–V4); PERMANOVA (Bray–Curtis) and multilevel PCA DESeq2 differential abundance; targeted analysis of *Akkermansia muciniphila*	44 metformin-treated T2DM adults 24 dapagliflozin 10 mg + 20 gliclazide 30 mg (3 faecal samples missing in gliclazide arm)	No significant change in α-diversity (observed ASVs, Shannon, Faith’s PD) in either arm No treatment-associated shift in β-diversity; subject identity was the strongest covariate of composition (PERMANOVA) Small overall temporal shift in composition (multilevel PCA, *p* = 0.02) not attributable to either drug No differentially abundant taxa identified; baseline composition did not predict metabolic response *A. muciniphila*: unchanged (despite prior murine signal under dapagliflozin)	No genus- or family-level enrichment or depletion attributable to dapagliflozin or gliclazide Inter-individual variability dominated over treatment effect *A. muciniphila* unaffected	First human evidence that SGLT2 inhibition does not measurably alter colonic microbiome composition when added to metformin Dapagliflozin and gliclazide similarly improved HbA1c with divergent metabolic profiles (↓ insulin/BMI vs. ↑ insulin/BMI), none mediated by faecal microbiota remodelling Background metformin may mask small drug-specific effects
Deng et al., 2022 [91]	Open-label RCT (3 months) Treatment-naïve T2DM with CVD risk factors 16S rRNA sequencing (V3–V4) + untargeted plasma metabolomics (UHPLC-QTOF-MS) Comparative head-to-head against metformin (active comparator) LEfSe differential abundance; aPCoA (unweighted UniFrac); multi-omics correlation	76 treatment-naïve T2DM adults with ≥1 CVD risk factor Empagliflozin 10 mg/day (n = 40) vs. metformin 1700 mg/day (n = 36) 91% completed 3-month follow-up	Empagliflozin significantly reshaped gut microbiota after 1 month; alteration maintained through 3 months Enriched SCFA-producing commensals and depleted pro-inflammatory/pathobiont taxa Distinct microbiota signature vs. metformin, with overlapping benefits on butyrogenic genera Microbiota changes correlated with plasma metabolomic shifts (↑ sphingomyelin; ↓ uric acid, cis-aconitate, glycochenodeoxycholate) α-diversity (Shannon index, observed ASVs) significantly increased in empagliflozin group but not metformin	↑ *Roseburia*, *Eubacterium*, *Faecalibacterium* (butyrate producers) ↑ *Ruminococcaceae*, *Lachnospiraceae* ↓ *Escherichia–Shigella*, *Bilophila*, *Hungatella* (pro-inflammatory/pathobiont taxa) 4 ASVs (*Blautia*, *Roseburia*, *Ruminococcaceae*, *Lachnospira*) shared between empagliflozin and metformin	First RCT evidence that empagliflozin remodels gut microbiota in a direction associated with cardiovascular and metabolic benefit Cardiovascular benefits of empagliflozin in T2DM may be partly mediated by favourable gut microbiota–metabolite remodelling Supports empagliflozin as a first-line option in T2DM patients with CVD risk factors Contrasts with van Bommel et al. (no effect when added to metformin), suggesting baseline metformin may obscure SGLT2i-specific microbiota effects

Abbreviations and symbols: ↑, increase/enrichment relative to comparator or baseline; ↓, decrease/depletion relative to comparator or baseline.

**Table 9:** Key clinical studies evaluating oral iron supplement effects on gut microbiota composition and nutritional outcomes.

Author, Year	Study Design	N	Key Microbiota Findings	Principal Taxa Affected	Clinical Implication
Zimmermann et al., 2010 [38]	Randomised controlled trial (6 months) School-based intervention in Ivorian children aged 6–14 years TGGE microbial profiling + targeted qPCR (enterobacteria, lactobacilli, bifidobacteria, *Bacteroides*, *Salmonella*) Faecal calprotectin; iron status indices (Hb, ferritin, sTfR, ZPP)	139 schoolchildren (60 in microbiota substudy) Iron-fortified biscuits (20 mg electrolytic Fe, 4×/week) vs. unfortified control	Iron fortification induced significant compositional dysbiosis vs. control (TGGE dissimilarity 32.3% vs. 15.0%, *p* < 0.0001) Significant ↑ enterobacteria (*p* < 0.005) and ↓ lactobacilli (*p* < 0.0001) by qPCR No change in total bacteria, *Bacteroides*, or *Bifidobacterium* ↑ faecal calprotectin in iron group (*p* < 0.01), correlating with enterobacterial expansion (r = 0.32, *p* < 0.05) Iron fortification ineffective: no improvement in iron status or anaemia despite ~1.5 g total Fe consumed	↑ Enterobacteriaceae (including commensal and pathogenic *E. coli*, *Salmonella*) ↓ *Lactobacillus* spp. (iron-independent protective commensals) Shift toward a pro-inflammatory community structure with elevated enterobacteria:lactobacilli ratio	First field RCT evidence that poorly absorbed fortification iron induces dysbiosis and gut inflammation in African schoolchildren Raises safety concerns about untargeted iron fortification in settings with high baseline enteropathogen exposure Supports screening-guided, targeted iron interventions rather than blanket population fortification
Jaeggi et al., 2015 [30]	Double-blind RCT (4 months) Kenyan weaning infants (6–10 months at baseline) 16S rRNA pyrosequencing + qPCR of pathogen virulence genes (EPEC, ETEC, EHEC, *Salmonella*, *C. difficile*, *C. perfringens*) Faecal calprotectin; diarrhoea incidence (active surveillance)	115 weaning infants (101 analysed) MNP with 2.5 mg Fe (NaFeEDTA) or 12.5 mg Fe (ferrous fumarate) vs. no-iron MNP control	↑ *Escherichia/Shigella* (*p* = 0.010) and *Clostridium* (*p* = 0.033) in +FeMNP vs. −FeMNP at endpoint Trend toward ↓ *Bifidobacterium* (*p* = 0.085); significant ↓ with 12.5 mg dose (*p* = 0.047) ↑ enterobacteria:bifidobacteria ratio (pyrosequencing *p* = 0.020; qPCR *p* = 0.008) ↑ pathogenic *E. coli* virulence genes (sum of 5 strains: *p* = 0.029) ↑ faecal calprotectin (*p* = 0.002); especially with 12.5 mg dose (*p* = 0.008) Trend toward ↑ diarrhoea in +12.5 mgFeMNP (27.3% vs. 8.3%, *p* = 0.092)	↑ Enterobacteriaceae (*Escherichia/Shigella*, pathogenic *E. coli* strains) ↑ *Clostridium* spp. ↓ *Bifidobacterium* (key protective infant commensal) ↓ *Roseburia* spp./*E. rectale* (butyrate producers; by qPCR, *p* = 0.020)	First well-powered RCT demonstrating iron-driven dysbiosis in African infants with functional pro-inflammatory consequences Untargeted iron fortification increases pathogen abundance and gut inflammation in populations with high baseline enteropathogen carriage Supports targeted, screening-based iron supplementation in vulnerable paediatric populations Highlights need for co-interventions (e.g., prebiotics) to mitigate iron-driven dysbiosis
Paganini et al., 2017 [44]	Double-blind factorial RCT (4 months) Kenyan weaning infants (6.5–9.5 months) 16S rRNA sequencing + qPCR for 10 enteropathogen virulence/toxin genes Plasma I-FABP (enterocyte damage); faecal calprotectin; faecal pH Respiratory tract infection surveillance	155 infants randomised to three arms: (1) Control MNP (no iron) (2) Fe MNP (5 mg Fe) (3) FeGOS MNP (5 mg Fe + 7.5 g GOS)	Anaemia reduced ~50% in both Fe and FeGOS groups (*p* < 0.001) Fe group: ↓ *Bifidobacterium* (*p* = 0.007 vs. FeGOS) and *Lactobacillus* (*p* = 0.006 vs. FeGOS); ↑ Clostridiales (*p* = 0.001 vs. FeGOS) FeGOS group: no significant differences vs. control in key taxa ↓ sum of pathogen VTGs in FeGOS vs. both control and Fe at 3 weeks (*p* < 0.01) ↑ I-FABP in Fe vs. control (*p* = 0.0498); no difference FeGOS vs. control ↑ RTI incidence in Fe group (87%) vs. control (75%, *p* = 0.024) and FeGOS (75%); ↓ RTIs over time in FeGOS (*p* = 0.013)	Fe group: ↓ *Bifidobacterium*, ↓ *Lactobacillus*, ↑ Clostridiales, ↑ Ruminococcaceae, Lachnospiraceae FeGOS group: preserved *Bifidobacterium* and *Lactobacillus* at control-equivalent levels ↓ pathogen VTGs (incl. EHEC stx2) in FeGOS vs. Fe and control	Combined low-dose iron–prebiotic (GOS) strategy maintains antianaemic efficacy while limiting microbiome disruption and enterocyte injury Reduces respiratory infection risk associated with iron-induced dysbiosis Proof-of-concept for combined iron–prebiotic nutritional strategies in paediatric populations at high enteropathogen risk 5 mg iron dose with GOS is efficacious and safer than standard 12.5 mg MNPs

Abbreviations and symbols: ↑, increase/enrichment relative to comparator or baseline; ↓, decrease/depletion relative to comparator or baseline.

**Table 10:** Proposed drug combination effects on gut microbiota in polypharmacy scenarios.

Drug Combination	Net Microbiota Effect	Proposed Mechanism	Clinical Consequence
PPI + NSAID	Synergistic: ↑↑ Enterobacteriaceae; ↑↑ gut permeability beyond either drug alone	Acid suppression → Gram-negative small bowel colonisation + COX inhibition → prostaglandin depletion → compounded barrier failure	↑↑ SIBO; ↑↑ small intestinal bleeding; ↑ CDI; substantially reduced colonisation resistance [24,76,77].
Metformin + PPI	Partial counteraction: Akkermansia expansion (metformin) partially offsets strict anaerobe depletion (PPI); oral taxa enrichment persists	Competing selective pressures; net result: metabolic benefit preserved, PPI dysbiosis attenuated but not eliminated	Glycaemic benefit maintained; CDI risk from PPI unchanged; attenuated but persistent dysbiosis [3,22,40,64].
Metformin + NSAID	Partially protective: metformin-expanded butyrate producers may blunt *F. prausnitzii* depletion; Akkermansia improves mucosal resilience	Butyrate → colonocyte fuel → improved barrier → attenuated NSAID damage; Akkermansia → tight junction upregulation	Reduced NSAID enteropathy in T2DM patients on metformin (observational evidence); prospective data lacking [66,103].
Statin + PPI	Partially antagonistic: statin enrichment of *F. prausnitzii* and Roseburia partially offsets PPI depletion	Statin anti-inflammatory effects attenuate PPI-driven mucosal vulnerability; bile acid modulation partially counteracts pH-mediated shifts	Theoretical attenuation of PPI dysbiosis in cardiovascular patients; empirical data needed [3,25,39].
Metformin + SGLT2i	Potentially synergistic beneficial: convergent Akkermansia enrichment through distinct pathways	Metformin → mucin secretion → Akkermansia niche; SGLT2i → reduced mucosal inflammation → anti-inflammatory taxa expansion	Enhanced microbiome-mediated glucose control; emerging clinical evidence supports dual benefit [28,64,91,92].
PPI + Oral iron	Compounding adverse: overlapping selection for Gram-negative pathobionts	PPI removes acid barrier + iron provides siderophilic growth advantage → ↑↑ Enterobacteriaceae	↑↑ infection susceptibility; ↑ SIBO; reduced colonisation resistance; common in elderly anaemic patients [3,29,30,53].
PPI + Metformin + NSAID (triple)	Complex, predominantly adverse: ↑↑↑ Proteobacteria; ↓↓↓ *F. prausnitzii*; ↓↓↓ Roseburia; metformin benefit overwhelmed	Summation and synergism of individual mechanisms; non-linear interactions	Substantially ↑ CDI, SIBO, enteric bleeding, systemic endotoxaemia; highest risk in elderly and frail patients [5,19,76,104].

Abbreviations and symbols: ↑, increase/enrichment relative to comparator or baseline; ↑↑, marked or synergistic increase beyond the effect of either drug alone; ↑↑↑, pronounced increase under combined (triple) exposure; ↓↓↓, pronounced depletion under combined (triple) exposure; →, leads to/results in (mechanistic or causal direction).

## Data Availability

No new data were created or analysed in this study. Data sharing is not applicable to this article.

## Abbreviations

The following abbreviations are used in this manuscript:

16S rRNA	16S ribosomal RNA
ACS	Acute coronary syndrome
ADR	Adverse drug reaction
AMPK	AMP-activated protein kinase
ASV	Amplicon sequence variant
AUC	Area under the curve
BMI	Body mass index
BSH	Bile salt hydrolase
CDI	*Clostridioides difficile* infection
CFU	Colony-forming units
COX	Cyclooxygenase
CVD	Cardiovascular disease
DDI	Drug–drug interaction
EHEC	Enterohaemorrhagic *Escherichia coli*
EPEC	Enteropathogenic *Escherichia coli*
ETEC	Enterotoxigenic *Escherichia coli*
FDR	False discovery rate
FGFP	Flemish Gut Flora Project
FMT	Faecal microbiota transplantation
FXR	Farnesoid X receptor
GERD	Gastroesophageal reflux disease
GI	Gastrointestinal
GLP-1	Glucagon-like peptide-1
GLP-1RA	Glucagon-like peptide-1 receptor agonist
GOS	Galacto-oligosaccharides
GRADE	Grading of Recommendations, Assessment, Development and Evaluation
GUDCA	Glycoursodeoxycholic acid
HFD	High-fat diet
HMG-CoA	3-hydroxy-3-methylglutaryl-coenzyme A
I-FABP	Intestinal fatty acid-binding protein
IBD	Inflammatory bowel disease
IBS	Irritable bowel syndrome
LEfSe	Linear discriminant analysis effect size
MADDS	Microbiome-active drug delivery systems
MDR	Multidrug-resistant
MNP	Micronutrient powder
NSAID	Non-steroidal anti-inflammatory drug
OCT1	Organic cation transporter 1
OR	Odds ratio
OTU	Operational taxonomic unit
PCA	Principal component analysis
PCoA	Principal coordinate analysis
PD	Phylogenetic diversity (Faith’s PD)
PERMANOVA	Permutational multivariate analysis of variance
PICO	Population, Intervention, Comparison, Outcome
PICRUSt	Phylogenetic Investigation of Communities by Reconstruction of Unobserved States
PPI	Proton pump inhibitor
PRISMA	Preferred Reporting Items for Systematic reviews and Meta-Analyses
QMP	Quantitative microbiome profiling
qPCR	Quantitative polymerase chain reaction
RCT	Randomised controlled trial
RoB 2	Risk of Bias 2 tool
ROS	Reactive oxygen species
RT-qPCR	Reverse transcription quantitative polymerase chain reaction
SCFA	Short-chain fatty acid
SGLT1	Sodium-glucose co-transporter 1
SGLT2	Sodium-glucose co-transporter 2
SGLT2i	Sodium-glucose co-transporter 2 inhibitor
SIBO	Small intestinal bacterial overgrowth
T2DM	Type 2 diabetes mellitus
TGGE	Temperature gradient gel electrophoresis
TUDCA	Tauroursodeoxycholic acid
UPLC-MS/MS	Ultra-performance liquid chromatography–tandem mass spectrometry
UPLC-QTOF-MS	Ultra-performance liquid chromatography quadrupole time-of-flight mass spectrometry
VTG	Virulence/toxin gene
